# Photocatalyzed synthesis of isochromanones and isobenzofuranones under batch and flow conditions

**DOI:** 10.3762/bjoc.13.143

**Published:** 2017-07-25

**Authors:** Manuel Anselmo, Lisa Moni, Hossny Ismail, Davide Comoretto, Renata Riva, Andrea Basso

**Affiliations:** 1 Università degli Studi di Genova, Dipartimento di Chimica e Chimica Industriale, Via Dodecaneso 31, Genova, Italy

**Keywords:** flow chemistry, heterocycles, multicomponent reactions, photocatalysis, photo-Meerwein arylation–addition

## Abstract

Photocatalyzed reactions of 2-(alkoxycarbonyl)benzenediazonium tetrafluoroborates with various alkenes afforded isochromanones in good yields, according to a mechanism that was investigated. The advantage of using highly soluble esters rather than carboxylic acids as starting compounds became evident when the reactions were performed under flow conditions. On the other hand, when 2-vinylbenzoic acid derivatives were employed as reagents, isobenzofuranones were obtained together with unprecedented benzo[*e*][1,3]oxazepin-1(5*H*)-ones, with the latter derived from incorporation of the solvent (acetonitrile).

## Introduction

**Scheme 1:**
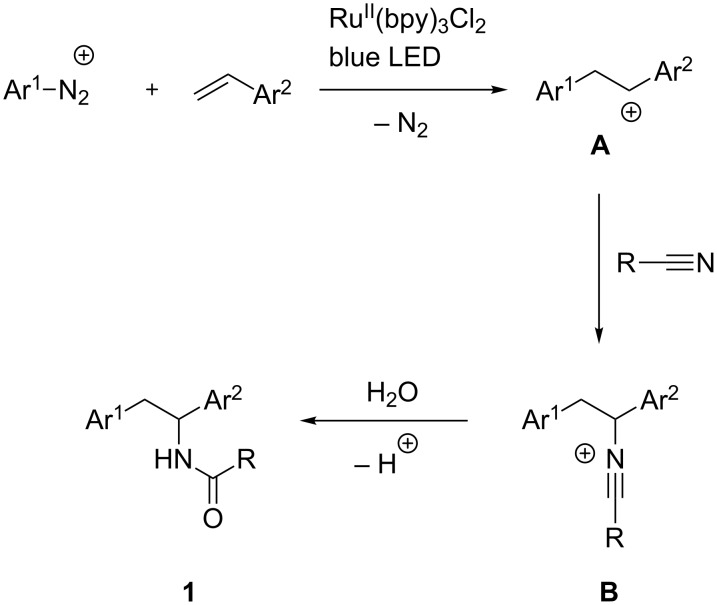
Photo-Meerwein reaction leading to amides.

Photoinduced multicomponent reactions are currently receiving remarkable attention [1]. Indeed, the possibility to obtain multiple-bond forming reactions under clean and mild conditions is nowadays one of the main targets of green chemistry approaches thus arousing our interest [2]. In 2014 König reported a photo-Meerwein reaction between arenediazonium salts and styrenes to afford, upon intervention of the nitrile solvent and water, amides of formula **1** (Scheme 1) [3]. According to the proposed mechanism, the carbocationic intermediate **A**, deriving from the corresponding radical, undergoes a nucleophilic attack by the nitrile, generating nitrilium ion **B** first, and subsequently evolves to the amide **1**, according to a Ritter-type reaction.

**Figure 1:**
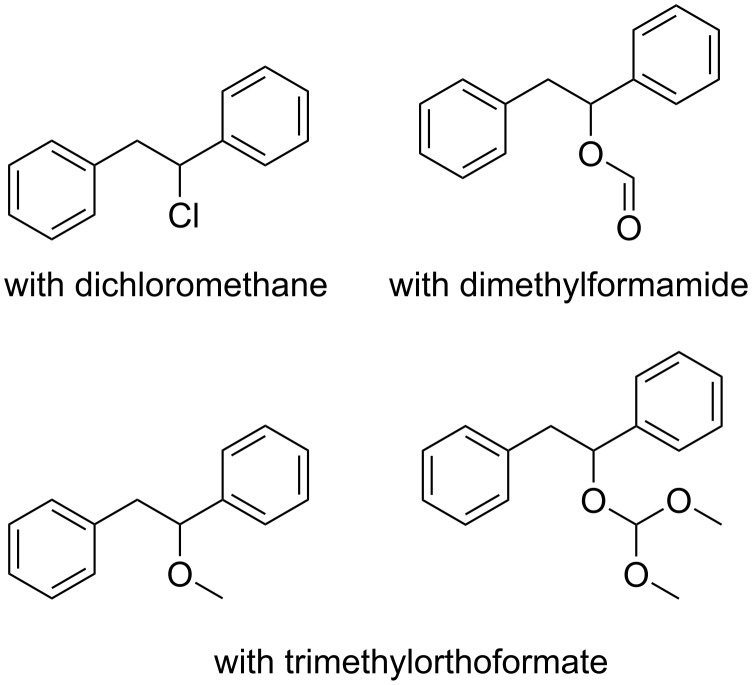
Products detected in the reaction mixtures during attempts to intercept the radical/cationic intermediate with various nucleophiles (solvents employed are reported).

Based on our experience on multicomponent reactions, we postulated that intermediate **A** could be intercepted by other nucleophiles (such as isocyanides, carboxylates, etc.), in the case the attack by the solvent could be prevented. Unfortunately, reactions performed in inert solvents with isocyanides, carboxylic acids, and mixtures of both, alcohols or amines never succeeded in affording the desired multicomponent adducts. It was rather curious instead to find out that many solvents or additives would attack the radical/cationic intermediate while reactants rationally and appositely selected would not. Some products unexpectedly obtained during various attempts are shown in Figure 1. In some cases also 1,2-diphenylethanol was isolated, although the origin of water was not clear (possibly trapped by the diazonium salts during crystallization since, due to their potential explosivity in the dry state, they were not dried under vacuum).

At this stage we postulated that **A** could be more favourably attacked in an intramolecular fashion, with the nucleophilic group appropriately displayed by one of the reagents. By inspection of the literature data, we were surprised to find only one example where benzothiophenes were obtained by reacting *o*-methylthioarenediazonium salts with alkynes under green light irradiation [4].

We therefore designed and synthesized reagents bearing ester or carboxylic functions in a convenient position, for a possible intramolecular attack, to investigate their reactivity. While working on this project, König and Fagnoni reported the photoredox-catalyzed reaction of 2-carboxybenzenediazonium salts with various alkenes [5], which prompted us to disclose our results in this communication.

## Results and Discussion

**Scheme 2:**
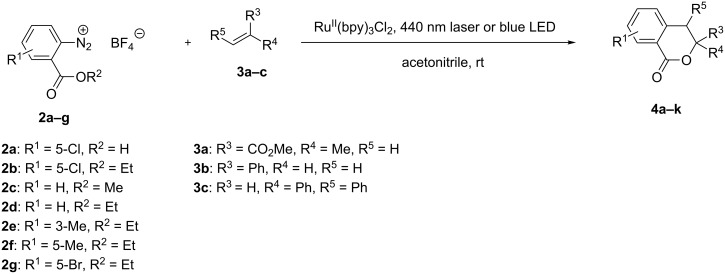
Reaction of *o*-alkoxycarbonyldiazonium salts with alkenes under Ru-photocatalyzed conditions.

When we reacted diazonium salt **2a** with methyl methacrylate (**3a**) in acetonitrile in the presence of a catalytic amount of Ru(bpy)_3_Cl_2_ under blue laser irradiation, we could isolate isochromanone **4a** in 72% yield (Scheme 2 and Table 1). Analogous results were obtained when the substitution pattern on both the diazonium salt and the alkene was varied, results that paralleled those reported by König and Fagnoni [5]. In our hands reactions were slower (6–8 h vs 2 h), probably due to the lower loading of photocatalyst (0.5% vs 2%) and excess of alkene (2 equiv vs 3 equiv) employed; moreover, the use of an inert atmosphere did not lead to significant improvements. No differences were observed when the 440 nm laser was replaced by blue LEDs (maximum wavelength 455 nm). Diazonium salts were partially insoluble in the reaction mixture, which became completely homogeneous only upon completion of the reaction. Having in mind to adapt this synthetic approach to flow conditions, partial insolubility of the reagents implied a serious drawback.

**Table 1:** Results of the reaction between diazonium salts **2** and alkenes **3**.

Entry	Diazonium salt	Alkene	Product	Yield^a^

1	**2a**	**3a**	**4a**	72%
2	**2b**	**3a**	**4a**	71%
3	**2b**	**3b**	**4b**	89%
4	**2d**	**3a**	**4c**	59%
5	**2d**	**3b**	**4d**	60%
6	**2e**	**3a**	**4e**	22%
7	**2e**	**3b**	**4f**	39%^b^
8	**2f**	**3a**	**4g**	59%
9	**2f**	**3b**	**4h**	50%
10	**2g**	**3a**	**4i**	53%
11	**2g**	**3b**	**4j**	34%
12	**2c**	**3c**	**4k**	19%

^a^Reactions were performed by mixing benzenediazonium tetrafluoroborates **2** (1 mmol), alkenes **3** (2 equiv) and Ru(bpy)_3_Cl_2_ (0.005 equiv) in CH_3_CN (4 mL). The reactions were irradiated with 440 nm LEDs for 6–8 h. Yields refer to isolated products after chromatographic purification; ^b^reaction was performed in acetonitrile/toluene 8:2.

According to our previous experience we know that the methyl/ethyl esters of diazonium salts derived from anthranilic acids were completely soluble in acetonitrile and other solvents such as acetone or toluene. Moreover, these salts were also more easily synthesized starting from the corresponding methyl/ethyl anthranilates. To our delight diazonium salt **2b** afforded isochromanone **4a** in 71% yield upon reaction with methyl methacrylate under the previously developed conditions. Similarly, compounds **4b–k** were synthesized under the same conditions, with yields ranging from moderate to good (Table 1). Also *trans*-stilbene reacted under the reaction conditions, but the 3,4-disubstituted isochromanone was isolated in only 19% yield. Noteworthy, compounds **4c**, **4d** and **4f**, whose synthesis was previously reported starting from carboxylic acids [5], were obtained with comparable yields.

**Scheme 3:**
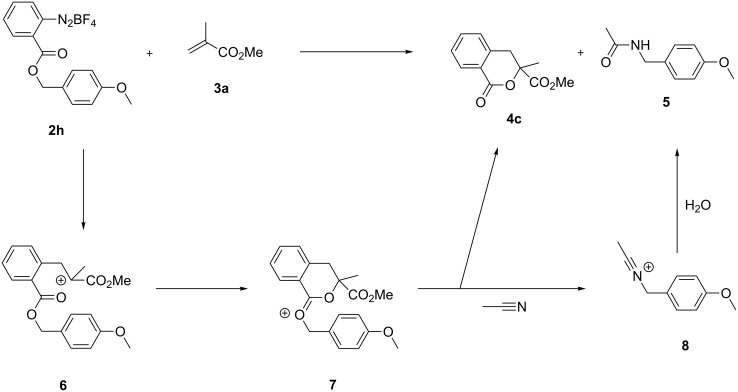
Proposed mechanism for the reaction of diazonium salt **2h** with methyl methacrylate (**3a**).

As no other detectable products were isolated from the reaction mixtures, we were intrigued by the fate of the alkyl group of the ester functionality that was not incorporated in the final products. Diazonium salt **2h**, bearing an alkyl group able to generate a more stable carbocationic species, was synthetized and reacted under standard conditions with alkene **3a**, affording isochromanone **4c** in 71% yield, together with an equimolar amount of acetamide **5** (Scheme 3). This outcome suggests that intermediate **6**, analogous to intermediate **A** depicted in Scheme 1, cyclizes through intramolecular nucleophilic attack of the carbonyl oxygen onto the positively charged carbon. The resulting oxonium ion **7** evolves to isochromanone **4c**, while the 4-methoxybenzyl carbocation is sufficiently long lived to react with acetonitrile and afford acetamide **5** upon reaction with traces of water. Interestingly enough, when the reaction was performed in acetone, compound **4c** was isolated in lower yield (60%) without any other detectable product. However, the TLC analysis of the crude material, after staining with cerium molybdate, displayed a plethora of red-colored spots typical for 4-methoxybenzyloxycarbonyl (MeOZ) cleavage with trifluoroacetic acid, thus suggesting the formation of a 4-methoxybenzyl carbocationic species.

**Scheme 4:**
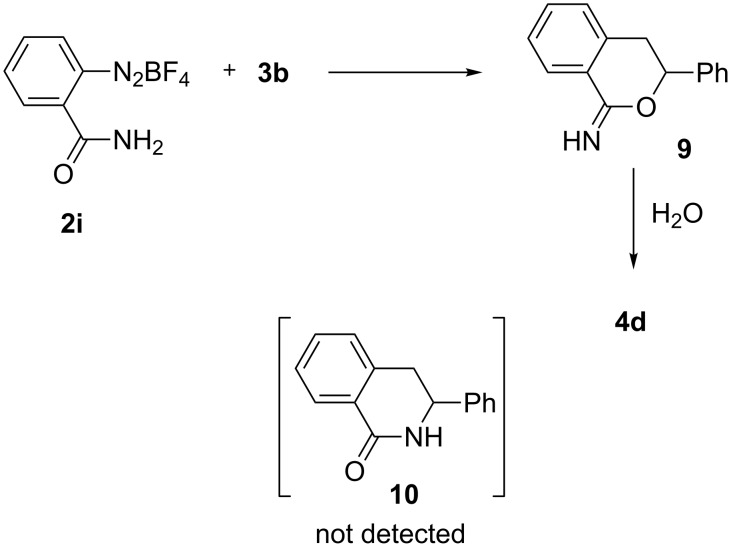
Reaction of 2-aminocarbonyldiazonium salt **2i** with styrene (**3b**).

In addition, when diazonium salt **2i** was reacted with styrene (**3b**), the formation of dihydroisoquinolinone **10** was not observed, while relatively unstable imidate **9** was detected. This observation supports our hypothesis that the nucleophilic attack to the intermediate **A** is taken by the carbonyl oxygen. Compound **9** immediately afforded isochromanone **4d** (60%) upon exposure to water (Scheme 4). Careful control of the isolation procedures (see Supporting Information File 1) allowed us to isolate **9** and identify it through GC–MS and ^1^H NMR experiments (the compound, however, partially decomposed during ^13^C NMR analysis).

The interest of both academia and industry in flow chemistry has recently increased. In particular, photochemical reactions performed under continuous flow have been proven particularly effective [6], as the reduced size of the reaction channels allows for a more efficient penetration of light through the reaction mixture. In addition, the removal of products from the irradiated area can suppress secondary photoreactions, leading to an improvement in yield and purity of the desired products. One of the major limitations of micro- and meso-flow applications are solubility issues, as poor dissolution of reagents and/or products can result in clogging and blockage of the flow apparatus.

**Figure 2:**
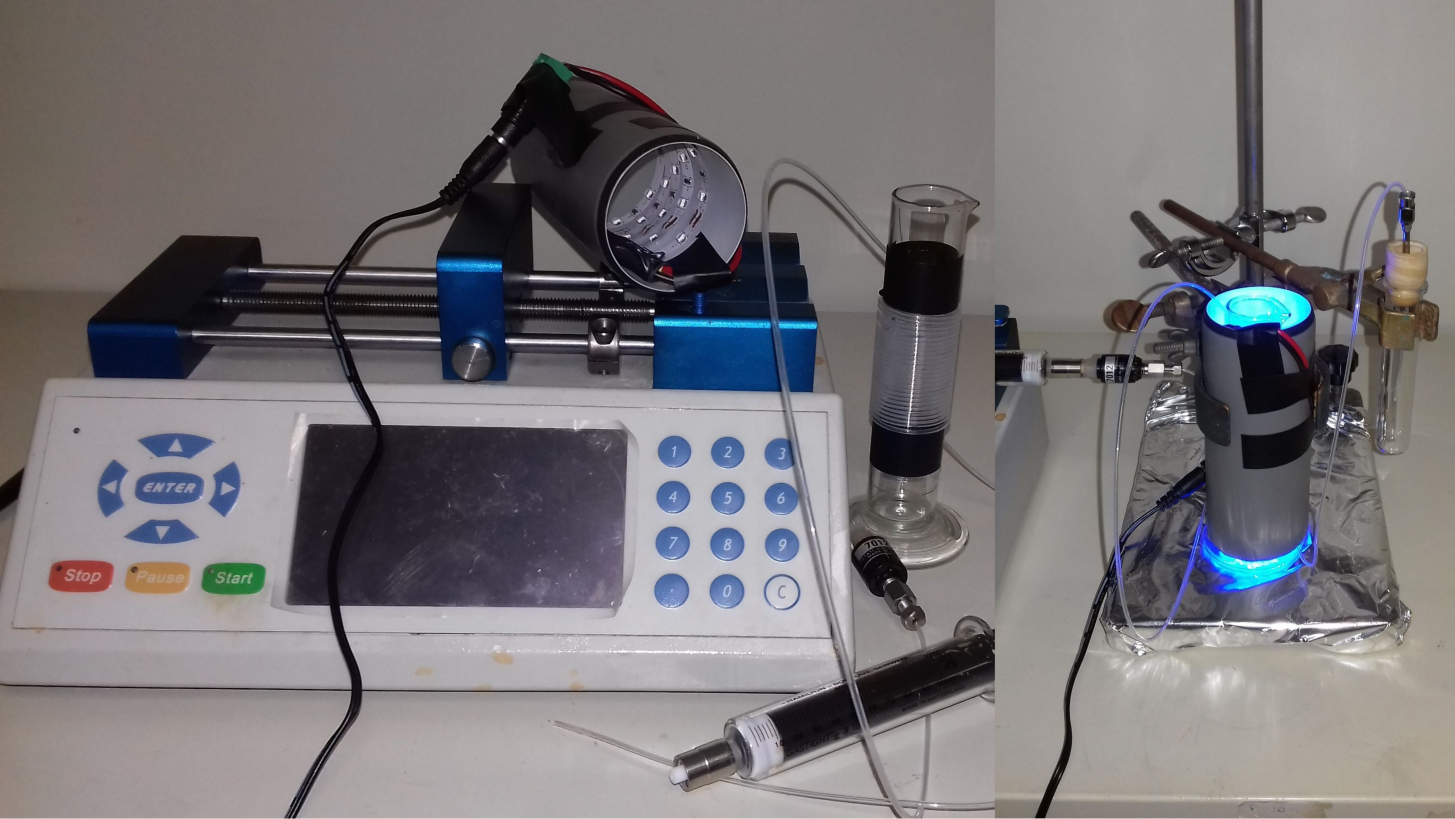
The meso-flow apparatus assembled in-house. The components are shown on the left, while the operating system is shown on the right.

Having solved the issue of partial insolubility of diazonium salts by replacing anthranilic acid derivatives with the corresponding esters and having demonstrated that an inert atmosphere was not essential, we therefore decided to test our synthetic methodology with an in house-made flow apparatus. The reaction coil was formed with 1 m of FEP tubing (internal diameter 0.8 mm) wrapped around a glass cylinder. Irradiation was obtained with strips of blue LEDs attached inside a hollow cylinder. The reagents were supplied by means of a single solution via a syringe pump (Figure 2).

The best results were obtained with a 130 mM solution of the diazonium salt in acetonitrile, 2 equiv of alkene and 0.5% of catalyst, and a residence time in the reaction coil (1 mL) of 10 h. Although nitrogen evolution started within the first few minutes of irradiation, shorter residence times resulted in lower conversions, as shown in Table 3 for model compound **4c** (with slower flow rates the diazonium salt started to decompose in the supplying syringe, as demonstrated by formation of nitrogen bubbles). All reagents were supplied by the same syringe, which was kept shielded from the light source. In fact, we verified that no reaction was taking place in the dark, even prolonging the experiment for several hours.

**Table 2:** Yield comparison at different flow rates.

Flow rate	Residence time	Yield

0.5 mL/h	2 h	41%
0.3 mL/h	3.3 h	53%
0.1 mL/h	10 h	71%

Although in the past we observed improvements in yields and purities switching from batch to flow conditions [7], in this case the results were not significantly better, as shown in Table 3.

**Table 3:** Comparison of yields obtained in batch and under flow conditions.

Compound	Batch	Flow^a^

**4c**	59%	71%
**4d**	60%	53%
**4e**	22%	29%
**4i**	53%	73%
**4k**	19%	20%

^a^Reactions under flow conditions were performed with benzendiazonium tetrafluoroborate **2** (1 equiv), alkene **3** (2 equiv) and Ru(bpy)_3_Cl_2_ (0.005 equiv) in CH_3_CN (0.13 M with respect to the diazonium salt).

**Scheme 5:**
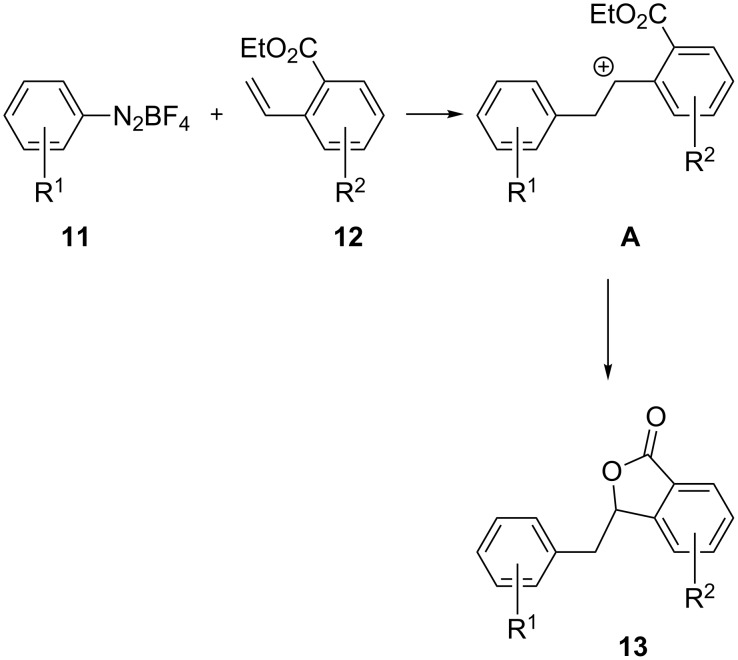
Reaction of diazonium salts **11** with styrenes **12**. The nucleophilic attack to intermediate **A** is given by the ester moiety displayed by **12**.

Having set up the conditions for the synthesis of isochromanones, we moved to explore the possibility to obtain isobenzofuranones **13** by introducing an ester functional group in the alkene building block, according to the pathway depicted in Scheme 5.

**Scheme 6:**
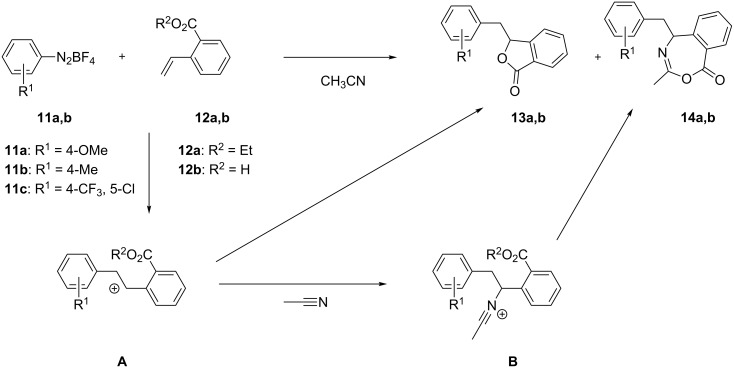
Proposed mechanism for the formation of benzo[*e*][1,3]oxazepin-1(5*H*)-one **14**.

The reaction was initially tested with 4-methoxybenzenediazonium tetrafluoroborate (**11a**) and 2-ethoxycarbonylstyrene (**12a**) under the conditions previously developed for isochromanones. The reaction, together with isobenzofuranone **13a** (31% yield), unexpectedly afforded also compound **14a** in 17% yield (Scheme 6). Benzoxazepinones **14**, the structure of which is unprecedented in the literature to the best of our knowledge, were the result of a three component reaction between the diazonium salt, ethoxycarbonylstyrene and acetonitrile. The postulated mechanism takes the fact into consideration that cyclization of intermediate **A** to isobenzofuranone is possibly hampered by steric strain. The attack of acetonitrile therefore becomes competitive (this is not observed in the case of isobenzochromanones). Nitrilium ion **B** is then intramolecularly intercepted by the ester moiety and a seven-membered ring is formed, with loss of an ethyl group.

Aiming to explore the selectivity of the reaction towards these two products, we conducted some experiments varying in particular the operating temperature and the solvent employed. The results are shown in Table 4. We observed that an improvement of the yield (62%) could be achieved by lowering the reaction temperature to −15 °C, even though the effect on the selectivity among the two products remained unchanged (Table 4, entry 2). When acetone was used as solvent, isobenzofuranone **13a** was selectively obtained in 40% yield (Table 4, entry 3). In order to test the reactivity of free acids as well, we submitted 2-vinylbenzoic acid (**12b**) to our reaction conditions, achieving an overall 41% yield and no selectivity among the two products (Table 4, entry 4). Having these results in hand, we have been surprised to discover a quite opposite selectivity when benzenediazonium tetrafluoroborate **11b** was reacted under the same conditions, giving a lower overall yield (43%) but with an opposite selectivity (3:1 in favour of the benzoxazepinone, Table 4, entry 5). When the same reaction was conducted with acetone as solvent, isobenzofuranone **13b** was selectively isolated with 34% yield (Table 4, entry 6). The higher selectivity in favour of benzoxazepinone **14b** has also been confirmed by the reaction of **11b** with 2-vinylbenzoic acid (**12b**), affording the major product **14b** in 41% yield together with traces of isobenzofuranone **13b** (Table 4, entry 7).

**Table 4:** Results of the reaction between diazonium salts **11** and alkenes **12**.

Entry	Diazonium salt	Alkene	Conditions	Product ratio	Yield^a^

1	**11a**	**12a**	CH_3_CN, rt	**13a/14a** 65:35	48%
2	**11a**	**12a**	CH_3_CN, −15 °C	**13a/14a** 68:32	62%
3	**11a**	**12a**	acetone, rt	**13a/14a** 100:0	40%
4	**11a**	**12b**	CH_3_CN, rt	**13a/14a** 46:54	41%
5	**11b**	**12a**	CH_3_CN, −15 °C	**13b/14b** 23:77	43%
6	**11b**	**12a**	acetone, rt	**13b/14b** 100:0	34%
7	**11b**	**12b**	CH_3_CN, rt	**13b/14b** <5:95	41%
8	**11c**	**12a**	CH_3_CN, rt		traces
9	**11a**	**12a**	CH_3_CN, flow	**13a/14a** 68:32	38%

^a^Reaction were performed by mixing benzenediazonium tetrafluoroborate **11** (1 mmol), alkene **12** (1.2 equiv) and Ru(bpy)_3_Cl_2_ (0.005 equiv) in CH_3_CN (4 mL). The reactions were irradiated with 440 nm LEDs for 6–8 h. Overall yields are referred to isolated products after chromatographic purification.

Apparently, the electronic effects of the substituents of the diazonium salt play a major role, especially when considering the reactivity of benzenediazonium tetrafluoroborate **11c** which, upon reaction with 2-ethoxycarbonylstyrene (**12a**), furnished only traces of the products, showing a dramatic dependence of the reaction course from the substrates employed.

The reaction of **11a** and **12a** was also performed under flow conditions, with no apparent improvement (Table 4, entry 9). These results will be the starting point for future investigations on the topic by us.

**Scheme 7:**
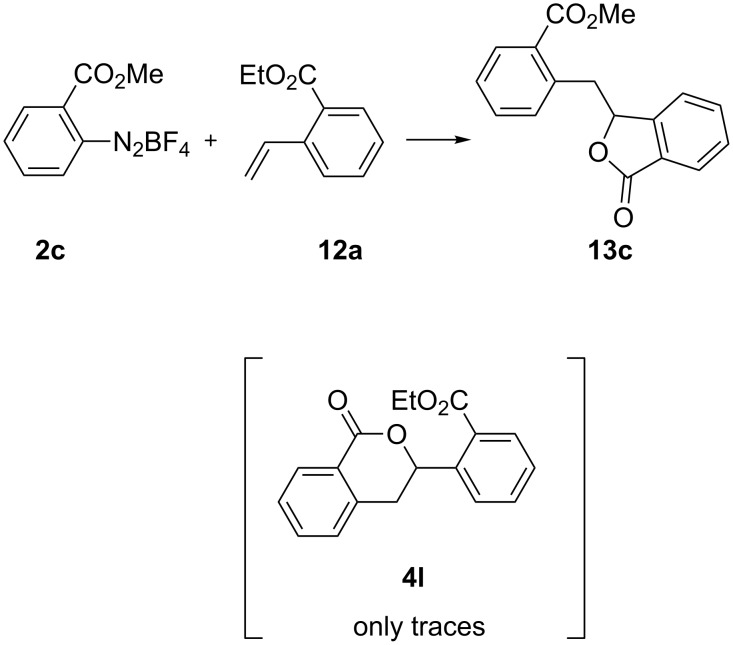
Investigation of the selectivity of the photochemically induced cyclization.

We were expecting to find further demonstration that cyclization to isochromanone would be favored with respect to benzofuranone from the reaction of compound **2c** with **12a**. In this case both substrates bear an ester functionality and in principle both six- and five-membered rings could be obtained. Unexpectedly, upon exposure to standard reaction conditions, isobenzofuranone **13c** was isolated in 58% yield, while only traces of isochromanone **4l** were detected in the crude material (Scheme 7). This finding contradicted our hypothesis that the formation of isochromanones was preferred, and will be the subject of further investigations.

## Conclusion

Benzopyranones are widespread in nature, being found in a wide variety of organisms, including fungi, and are endowed with a broad-spectrum of biological activities [8]. Also benzofuranones are found in nature, for example the paecilomycins/aigialomycin group of natural products co-exists with their corresponding benzochromanone isomers [9]. In this paper we described a photochemical approach to these two classes of compounds, starting from benzenediazonium salts and alkenes, functionalized with an alkoxycarbonyl group. Depending on which substrate bears the additional functionality, selective synthesis of benzo-fused six- or five-membered rings can be achieved. We have demonstrated that the 5-membered ring formation is favored, when the two processes are competitive, and we have also shown that acetonitrile can act as a third component in the cyclization to oxazepinones. By employing alkoxycarbonyl groups instead of hydroxycarbonyl ones we have also solved solubility issues and have therefore been able to transfer our methodologies under flow conditions. We are currently investigating further advances in this field, and results will be reported in due course.

## Supporting Information

File 1 Experimental details and detailed spectroscopic data.
