# Supplementary material for: Photocatalyzed synthesis of isochromanones and isobenzofuranones under batch and flow conditions
Source: Beilstein J Org Chem. 2017 Jul 25;13:1456–62. doi: 10.3762/bjoc.13.143 (PMC5550801; doi:10.3762/bjoc.13.143)

Supporting Information

for

Photocatalyzed synthesis of isochromanones and isobenzofuranones under batch and flow conditions

Manuel Anselmo, Lisa Moni, Hossny Ismail, Davide Comoretto, Renata Riva and Andrea Basso*

Address: Università degli Studi di Genova, Dipartimento di Chimica e Chimica Industriale, Via Dodecaneso 31, Genova, Italy

Email: Prof. Andrea Basso - andrea.basso@unige.it

*Corresponding author

Experimental details and detailed spectroscopic data

Table of contents

General experimental methods S2
General procedures S3
Emission spectrum of blue LEDs S4
Analytical data S5
Copies of NMR spectra S15

General experimental methods.

NMR spectra were recorded at 300 MHz (^1H) and 75 MHz (^{13}C) and the chemical shifts (δ) are expressed in parts per million relative to tetramethylsylane (TMS) as internal standard (0.00 ppm). Coupling constants are reported in hertz. NMR acquisitions were performed at 295 K and $CDCl_3$ was used as solvent.

GC–MS analyses were carried out on a Hewlett Packard 5890 Series II, using a HP-1 column, coupled with a HP-5971A spectrometer (electron impact). Analysis conditions are as follows: flow (He) 0.9 mL/min; initial temperature 100 °C; initial time 2 min; gradient temperature 20 °C/min; final temperature 280 °C; final time 5 min.

HPLC–MS analyses were carried out on a Hewlett Packard 1100, using a Gemini C6-Phenyl (150x3mm) column, coupled with a Microsaic 4000 MiD Mass Spectrometer (electrospray). Analysis conditions are as follows: flow 0.34 mL/min; temperature 26 °C; solvent A: water + 0.1% formic acid; solvent B: acetonitrile + 0.1% formic acid; gradient: from 10% B to 100% B in 20 min; VWD 220 nm; MASS: FullScan 100-800 m/z ES^+, Tic voltage 750 V.

Photoinduced reactions were performed with blue LEDs (LED stripe GBC SMD 3528 INDOOR BLU 12Vcc) or LASER (CNI diode laser MDL-442, 40 mW power driven by power supply unit model PSU-III-LED. The output of laser is coupled to an optical fiber).

Reactions were monitored by TLC. TLC analyses were carried out on silica gel plates (thickness = 0.25 mm), viewed at UV (λ = 254 nm) and developed with Hanessian stain (dipping into a solution of $(NH_4)_4MoO_4\cdot 4H_2O$ (21 g) and $Ce(SO_4)_2\cdot 4H_2O$ (1 g) in H_2SO_4 (31 mL) and H_2O (469 mL) and warming).

Column chromatography was performed with the “flash” methodology using 220−400 mesh silica. Solvents employed as eluents and for all other routinary operations, as well as anhydrous solvents and all reagents used were purchased from commercial suppliers and employed without any further purification.

Benzenediazonium salts were prepared from the corresponding anilines according to literature procedures (i.e., Schuster G. B. et al. *J. Am. Chem. Soc.*, **1995**, *117*, 5206-5211).

General procedures

*Synthesis of isochromanones **4** under batch conditions*

A solution of benzendiazonium tetrafluoroborate **2** (1 mmol, 1 equiv) and $Ru(bpy)_3Cl_2$ (0.005 equiv) in CH_3CN (4 mL, 0.25 M with respect to the diazonium salt) was added with alkene **3** (2 mmol, 2 equiv) in a glass vial (Ø= 1.2 cm). The vial was exposed to the light generated by 440 nm LED bulbs for 6–8 hours. The solution was transferred into a round-bottomed flask, evaporated, and the crude was subsequently purified by column chromatography (PE/EA mixtures) to afford the final products as white solids/foams.

*Synthesis of isochromanones **4** under flow conditions*

Reactions in flow conditions were performed by means of a handmade mesoflow reactor consisting of a FEP tubing (internal Ø = 0.8 mm) wrapped up a glass cylinder (Ø = 2.5 cm) for a length corresponding exactly of 1 mL. The tube was equipped at one end with a connection for a gastight syringe (10 mL), fitting in a syringe pump. The wrapped cylinder was fitted inside a plastic cylinder (Ø = 4.5 cm) covered inside with LED bulbs (440 nm, model of the stripe).

A solution of benzendiazonium tetrafluoroborate **2** (1 equiv) and $Ru(bpy)_3Cl_2$ (0.005 eq) in CH_3CN (0.13 M with respect to the diazonium salt) was added with alkene **3** (2 equiv). The resulting clear solution was collected inside a 10 mL gastight syringe and pumped inside the flow reactor by means of a syringe pump at 0.1 mL/h. A fixed volume of the reacted solution was collected in a shielded round-bottomed flask, evaporated, and purified by column chromatography (PE/EA mixtures) to afford the final products as white solids/foams.

*Synthesis of isobenzofuranones **13** and benzoxazepinones **14** under batch conditions*

A solution of benzendiazonium tetrafluoroborate **11** (1 mmol, 1 equiv) and $Ru(bpy)_3Cl_2$ (0.005 equiv) in CH_3CN (4 mL, 0.25 M with respect to the diazonium salt) was added with alkene **12** (1.2 mmol, 1.2 equiv) in a glass vial (Ø = 1.2 cm). The vial was exposed to the light generated by 440 nm LED bulbs for 6–8 hours. The solution was transferred into a round-bottomed flask, evaporated, and the crude was subsequently purified by column chromatography (PE/EA mixtures) to afford the final products as white solids/foams.

*Synthesis of isobenzofuranones **13** and benzoxazepinones **14** under flow conditions*

The same conditions applied for the synthesis of isochromanones **4** were applied.

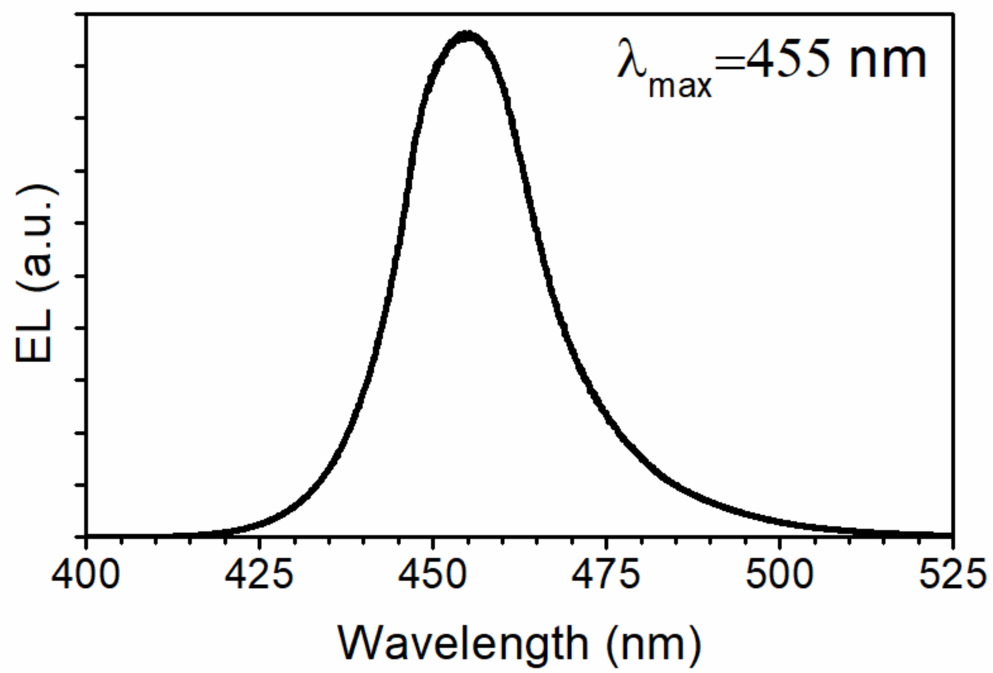
λ_{max}=455 nm
EL (a.u.)
400
425
450
475
500
525
Wavelength (nm)

Analytical data

*Methyl 7-chloro-3-methyl-1-oxoisochroman-3-carboxylate (**4a**)*

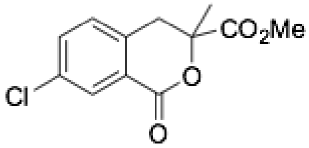

White solid, m.p.= 82.9-84.2°C

R_f = 0.22 (PE:EtOAc=4:1)

^{1}H NMR: 8.01 (d, *J* = 2.2, 1H Ar), 7.46 (dd, *J* = 8.2, 2.2, 1H Ar), 7.16 (d, *J* = 8.2, 1H Ar), 3.60 (s, 3H), 3.35 (d, *J* = 16.5, 1H), 3.16 (d, *J* = 16.5, 1H), 1.73 (s, 3H).

^{13}C NMR: 171.94, 163.12, 134.59, 134.14, 134.09, 129.87, 129.10, 126.09, 82.12, 53.24, 36.53, 24.96.

HPLC-MS: 13.0 min, 257 (255) $[M+H]^+$.

GC-MS: 7.2 min, 256 (2, M, ^{37}Cl), 254 (6, M, ^{35}Cl), 197 (30, ^{37}Cl), 195 (30, ^{35}Cl), 169 (12, ^{37}Cl), 167 (40, ^{35}Cl), 124 (11), 89 (25), 63 (13), 43 (73).

*7-Chloro-3-phenylisochroman-1-one (**4b**)*

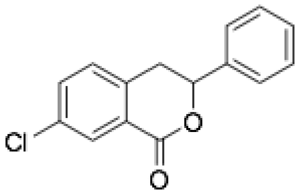

White solid, m.p.= 139.2-141.5

R_f = 0.44 (PE:EtOAc=4:1)

^{1}H NMR: 8.12 (d, *J* = 2.1, 1H), 7.53 (dd, *J* = 8.1, 2.1, 1H), 7.49 – 7.35 (m, 5H), 7.24 (d, *J* = 8.3, 1H), 5.53 (dd, X part of ABX system, *J* = 12.3, 3.1, 1H), 3.30 (dd, A part of ABX system, *J* = 16.5, 12.3, 1H), 3.13 (dd, B part of ABX system, *J* = 16.5, 3.1, 1H).

^{13}C NMR: 164.23, 138.24, 137.28, 134.07, 134.02, 130.31, 128.99, 128.94, 128.88, 126.72, 126.21, 80,12, 35.09.

HPLC-MS: 13.5 min, 261 (259) $[M+H]^+$.

GC-MS: 9.2 min, 260 (2, M, ^{37}Cl), 258 (5, M, ^{35}Cl), 154 (29, ^{37}Cl), 152 (100, ^{35}Cl), 126 (9, ^{37}Cl), 124 (29, ^{35}Cl), 89 (23).

*Methyl 3-methyl-1-oxoisochroman-3-carboxylate (**4c**)*

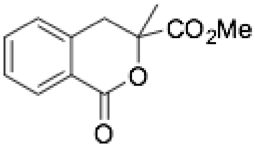

White solid, m.p.= 99.5-101.3

R_f = 0.29 (PE:EtOAc=4:1)

^{1}H NMR: 8.04 (d, *J* = 7.6, 1H Ar), 7.50 (td, *J* = 7.6, 1.2, 1H Ar), 7.35 (t, *J* = 7.6, 1H Ar), 7.19 (d, *J* = 7.6, 1H Ar), 3.58 (s, 3H), 3.38 (d, *J* = 16.4, 1H), 3.20 (d, *J* = 16.4, 1H), 1.73 (s, 3H).

^{13}C NMR: 172.28, 164.31, 136.33, 134.13, 130.16, 128.20, 127.57, 124.57, 82.02, 53.12, 37.07, 25.09.

HPLC-MS: 11.7 min, 221 $[M+H]^+$.

GC-MS: 6.5 min, 220 (2, M), 162 (11), 161 (100), 133 (64), 105 (14), 91 (11), 90 (24), 89 (22), 63 (11), 43 (45).

Analytical data are in accordance with those reported in the literature (ref. 5 of the manuscript)

*3-Phenylisochroman-1-one (**4d**)*

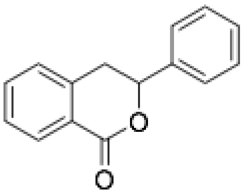

White solid, m.p.= 83.8-85-7

R_f = 0.55 (PE:EtOAc=4:1)

^{1}H NMR: 8.16 (d, *J* = 7.8, 1H Ar), 7.57 (td, *J* = 7.5, 1.0, 1H Ar), 7.49-7.35 (m, 6 H Ar), 7.29 (d, *J* = 7.6, 1H Ar), 5.56 (dd, X part of ABX system, *J* = 11.9, 4.6, 1 H), 3.35 (dd, A part of ABX system, *J* = 15.8, 11.9, 1 H), 3.13 (dd, B part of ABX system, *J* = 15.8, 4.6, 1H).

^{13}C NMR: 165.46, 139.05, 138.66, 134.05, 130.55, 128.81, 128.78, 128.01, 127.48, 126.24, 125.24, 80.09, 35.74.

HPLC-MS: 12.8 min, 225 $[M+H]^+$.

GC-MS: 8.4 min, 224 (9, M), 178 (6), 119 (18), 118 (100), 90 (68), 89 (26), 77 (13), 63 (10), 51 (12).

Analytical data are in accordance with those reported in the literature (ref. 5 of the manuscript)

*Methyl 3,5-dimethyl-1-oxoisochroman-3-carboxylate (**4e**)*

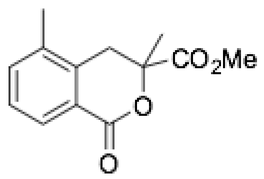

White solid, m.p.= 69.8-72.4

R_f = 0.30 (PE:EtOAc=4:1)

^{1}H NMR: 7.95 (d, *J* = 7.7, 1H Ar), 7.38 (d, *J* = 7.7, 1H Ar), 7.27 (t, *J* = 7.7, 1H Ar), 3.61 (s, 3H), 3.47 (d, *J* = 16.6, 1H), 2.99 (d, *J* = 16.6, 1H), 2.31 (s, 3H), 1.77 (s, 3H).

^{13}C NMR: 172.63, 164.75, 135.50, 135.49, 135.11, 128.08, 127.60, 124.62, 81.56, 53.20, 34.23, 25.37, 19.00.

HPLC-MS (ESI+): 13.6 min, 235 $[M+H]^+$.

GC-MS: 6.8 min, 234 (4, M), 175 (100), 147 (39), 105 (10), 104 (11), 103 (12) 78 (11), 77 (13), 43 (28).

*5-Methyl-3-phenylisochroman-1-one (**4f**)*

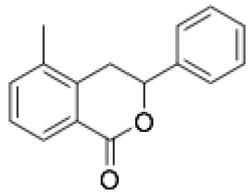

White solid, m.p.= 137.1-139.4

R_f = 0.60 (PE:EtOAc=4:1)

^{1}H NMR: 8.03 (dd, *J* = 7.7, 0.6, 1H Ar), 7.52-7.26 (m, 7H Ar), 5.52 (dd, *J* = 9.8, 5.6, 1H), 3.14 (m, 2H), 2.33 (s, 3H).

^{13}C NMR: 165.87, 138.95, 137.66, 135.42, 135.25, 128.83, 128.80, 128.41, 127.40, 126.31, 125.22, 79.53, 33.00, 19.04.

HPLC-MS: 14.4 min, 239 $[M+H]^+$.

GC-MS: 9.0 min, 238 (2, M), 132 (100), 104 (33), 103 (13), 78 (15), 77 (16).

Analytical data are in accordance with those reported in the literature (ref. 5 of the manuscript)

*Methyl 3,7-dimethyl-1-oxoisochroman-3-carboxylate (**4g**)*

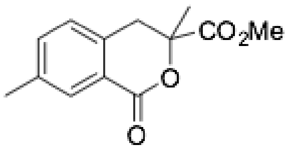

Foam

R_f = 0.19 (PE:EtOAc=5:1)

^{1}H NMR: 7.86 (s, 1H Ar), 7.30 (dd, *J* = 7.7, 1.4, 1H Ar), 7.08 (d, *J* = 7.7, 1H Ar), 3.58 (s, 3H), 3.33 (d, *J* = 16.3, 1H), 3.15 (d, *J* = 16.3, 1H), 2.34 (s, 3H), 1.72 (s, 3H).

^{13}C NMR: 172.40, 164.58, 138.08, 134.96, 133.35, 130.39, 127.44, 124.33, 82.09, 53.08, 36.76, 25.07, 21.08.

HPLC-MS: 12.2 min, 235 $[M+H]^+$.

GC-MS: 6.9 min, 234 (7, M), 175 (100), 147 (51), 104 (15), 103 (12), 78 (11), 77 (13) 43 (28).

*7-Methyl-3-phenylisochroman-1-one (**4h**)*

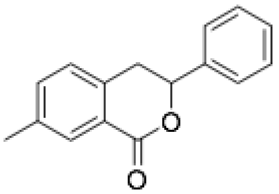

White solid, m.p.= 129.5-131.4°C

R_f = 0.35 (PE:EtOAc=6:1)

^{1}H NMR: 7.97 (s, 1H Ar), 7.50-7.36 (m, 6H Ar), 7.18 (d, *J* = 7.7, 1H Ar), 5.53 (dd, X part of ABX system, *J* = 12.2, 2.8, 1H), 3.29 (dd, A part of ABX system, *J* = 18.0, 12.2, 1H), 3.10 (dd, B part of ABX system, *J* = 18.0, 2.8, 1H), 2.41 (s, 3H).

^{13}C NMR: 165.72, 138.81, 137.90, 136.12, 134.92, 130.78, 128.78, 128.72, 127.36, 126.25, 125.01, 80.20, 35.39, 21.18.

HPLC-MS: 13.4 min, 239 $[M+H]^+$.

GC-MS: 8.9 min, 238 (28, m), 178 (19), 132 (100), 104 (100), 78 (23), 77 (34).

*Methyl 7-bromo-3-methyl-1-oxoisochroman-3-carboxylate (**4i**)*

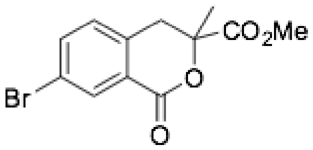

White solid, m.p.= 87.0-88.9°C

R_f = 0.34 (PE:EtOAc=4:1)

^{1}H NMR: 8.20 (d, *J* = 2.1, 1H Ar), 7.63 (dd, *J* = 8.1, 2.1, 1H Ar), 7.10 (d, *J* = 8.1, 1H Ar), 3.62 (s, 3H), 3.35 (d, *J* = 16.7, 1H), 3.15 (d, *J* = 16.7, 1H), 1.75 (s, 3H).

^{13}C NMR: 172.02, 163.05, 137.04, 135.08, 132.98, 129.31, 126.34, 121.95, 82.12, 53.35, 36.67, 25.08.

HPLC-MS: 15.3 min, 301 (299) $[M+H]^+$.

GC-MS: 7.8 min, 300 (9, M, ^{81}Br), 298 (9, M, ^{79}Br), 241 (88), 239 (90), 213 (31), 211 (33), 170 (11), 168 (11), 132 (24), 89 (43), 43 (100).

*7-Bromo-3-phenylisochroman-1-one (**4j**)*

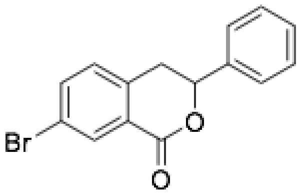

White solid, m.p.= 141.5-143.3°C

R_f = 0.16 (PE:EtOAc=10:1)

^{1}H NMR: 8.28 (d, *J* = 2.0, 1H Ar), 7.68 (dd, *J* = 8.2, 2.2, 1H Ar), 7.48-7.37 (m, 5H Ar), 7.18 (d, *J* = 8.2, 1H Ar), 5.55 (dd, X part of ABX system, *J* = 12.3, 2.7, 1H), 3.28 (dd, A part of ABX system, *J* = 16.5, 12.3, 1H), 3.12 (dd, B part of ABX system, *J* = 16.5, 2.7, 1H).

^{13}C NMR: 164.11, 138.20, 137.76, 136.95, 133.28, 129.21, 128.95, 128.88, 126.94, 126.20, 121.67, 80.06, 35.16.

HPLC-MS: 16.2 min, 305 (303) $[M+H]^+$.

GC-MS: 9.7 min, 304 (7, M, ^{81}Br), 302 (7, M, ^{79}Br), 198 (94, ^{81}Br), 196 (100, ^{79}Br), 170 (26, ^{81}Br), 168 (27, ^{79}Br), 89 (41), 77 (14), 63 (18).

*trans 3,4-Diphenylisochroman-1-one (**4k**)*

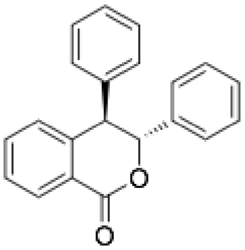

White solid, m.p.= 135.1-137.2

R_f = 0.43 (PE:EtOAc=6:1)

^{1}H NMR: 8.24 (dd, *J* = 7.4, 1.7, 1H Ar), 7.50 (td, *J* = 7.4, 1.7, 1H Ar), 7.47-7.41(m, 1H Ar), 7.27-7.14 (m, 8H Ar), 7.03-7.00 (m, 2H Ar), 6.92 (d, *J* = 7.4,1H Ar), 5.64 (d, *J* = 10.0, 1H), 4.51 (d, *J* = 10.0, 1H).

^{13}C NMR: 165.06, 142.67, 137.93, 137.42, 134.20, 130.44, 129.71, 128.94, 128.58, 128.30, 128.15, 127.98, 127.80, 127.20, 125.17, 85.40, 51.11.

HPLC-MS: 18.1 min, 301 $[M+H]^+$.

Decomposes during GC-MS analysis

*N-(4-methoxybenzyl)acetamide (**5**)*

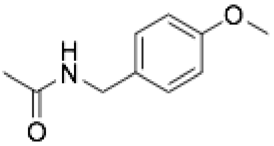

R_f = 0.13 (PE:EtOAc=1:1)

^{1}H NMR: 7.21 (d, *J* = 8.7, 2H Ar), 6.87 (d, *J* = 8.7, 2H Ar), 5.67 (broad s, 1H NH), 4.36 (d, *J* = 5.6, 2H), 3.80 (s, 3H), 2.01 (s, 3H).

Analytical data are in accordance with those reported in the literature (i. e. *Chem. Commun.* **2012**, *48*, 11626-11628)

*3-Phenylisochroman-1-imine (**9**)*

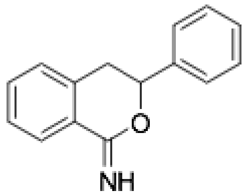

The crude mixture was concentrated under vacuum, no work-up was performed at this stage. A quick column cromatography was performed using EA/PE 1:4 with 5% Et_3N, affording the target compound as an oil.

Rf = 0.30 (PE:EtOAc=1:1)

^{1}H NMR: 8.23 (d, *J* = 7.5, 1H Ar), 7.55-7.33 (m, 8H, Ar + NH), 7.23 (d, *J* = 7.5 Hz, 1H Ar), 5.28 (dd, X part of ABX system *J* = 11.6, 2.9, 1H), 3.27 (dd, A part of ABX system *J* = 16.1, 11.6, 1H), 3.06 (dd, B part of ABX system *J* = 16.2, 2.9 Hz, 1H).

The compound degraded during ^{13}C NMR analysis

GC-MS: 8.1 min, 223 (7, M), 178 (7), 146 (13), 118 (17), 117 (80), 107 (100), 90 (19), 89 (18), 79 (15), 77 (18), 51(11).

*3-(4-Methoxybenzyl)isobenzofuran-1(3H)-one)(**13a**)*

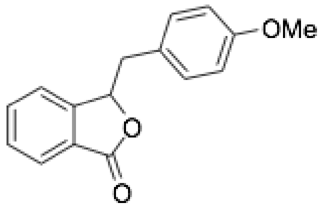

Foam

R_f = 0.40 (PE:EtOAc=4:1)

^{1}H NMR: 7.84 (d, *J* = 7.5, 1H Ar), 7.60 (td, *J* = 7.5, 1.1, 1H Ar), 7.48 (t, *J* = 7.5, 1H Ar), 7.17 (dd, *J* = 7.5 Hz, 0.8, 1H Ar), 7.13-7.09 (m, 2H Ar), 6.84-6.79 (m, 2H Ar), 5.65 (t, X part of ABX system, *J* = 6.3, 1H), 3.78 (s, 3H), 3.23 (dd, A part of ABX system, *J* = 14.1, 6.3, 1H), 3.10 (dd, B part of ABX system, *J* = 14.1, 6.3, 1H)

^{13}C NMR: 170.41, 158.81, 149.28, 133.78, 130.87, 129.24, 126.97, 126.42, 125.78, 122.4, 114.03, 81.50, 55.34, 40.03.

HPLC-MS: 13.0 min, 239 $[M+H]^+$.

GC-MS: 9.1 min, 254 (22, M), 133 (19), 121 (100), 78 (12) 77 (25).

Analytical data are in accordance with the literature (*J. Chem. Soc., Perkin Trans. 1* **1982**, 2819-2826)

*3-(4-Methylbenzyl)isobenzofuran-1(3H)-one (**13b**)*

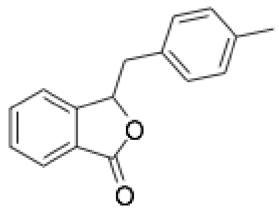

White solid, m.p.= 87.7-89.4 (*lit. 83-84*)

R_f = 0.47 (PE:EtOAc=4:1)

^{1}H NMR: 7.85 (d, *J* = 7.6, 1H Ar), 7.60 (td, *J* = 7.6, 1.2, 1H Ar), 7.49 (t, 7.6, 1H Ar), 7.16 (dd, *J* = 7.6, 0.8, 1H Ar), 7.10 (s, 4H Ar), 5.67 (t, X part of ABX system, *J* = 6.4, 1H), 3.26 (dd, A part of ABX system, *J* = 14.1 ,6.5, 1H), 3.10 (dd, B part of ABX system, *J* = 14.1, 6.5, 1H), 2.32 (s, 3H).

^{13}C NMR: 170.47, 149.35, 136.91, 133.80, 131.97, 129.72, 129.38, 129.28, 126.42, 125.82, 122.48, 81.51, 40.58, 21.23.

HPLC-MS: 14.5 min, 239 $[M+H]^+$.

GC-MS: 8.4 min, 238 (5, M), 133 (34), 106 (12), 105 (100), 77 (15).

Analytical data are in accordance with the literature (*Org. Lett.* **2009**, *11*, 4712-4715)

*Methyl 2-((3-oxo-1,3-dihydroisobenzofuran-1-yl)methyl)benzoate (**13c**)*

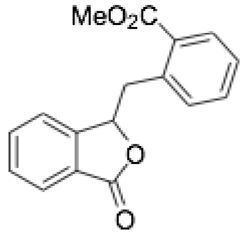

White solid, m.p.= 107.8-109.5 (*lit.* 111-112)

R_f = 0.36 (PE:EtOAc=4:1)

^{1}H NMR: 8.03 (dd, *J* = 8.1, 1.4, 1H Ar), 7.96-7.84 (m, 1H Ar), 7.66 (td, *J* = 7.5, 1.1, 1H Ar), 7.59-7.44 (m, 3H Ar), 7.40-7.34 (m, 2H Ar), 5.77 (dd, X part of ABX system, *J* = 9.0, 3.5, 1H), 3.95 (dd A part of ABX system, *J* = 13.7, 3.5, 1H), 3.94 (s, 3H), 3.12 (dd B part of ABX system, *J* = 13.7, 9.0, 1H).

^{13}C NMR: 170.62, 167.86, 149.99, 138.77, 134.03, 133.06, 132.64, 131.30, 129.31, 129.16, 127.52, 126.23, 125.73, 122.67, 82.12, 52.28, 40.65.

HPLC-MS: 13.4 min, 283 $[M+H]^+$.

GC-MS: 9.5 min, 282 (1, M), 250 (6), 149 (11), 133 (100), 105 (13), 77 (21), 51 (11).

Analytical data are in accordance with the literature (*Chem. Heterocycl. Compd.* **2012**, *47*, *1212-1224*)

*3-Methyl-5-(4-methoxybenzyl)benzo[e][1,3]oxazepin-1(5H)-one (**14a**)*

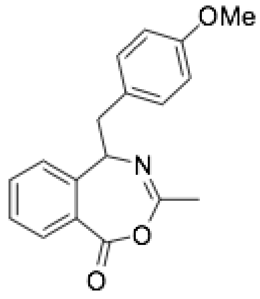

Foam

R_f = 0.45 (PE:EtOAc=4:1)

^{1}H NMR: 7.77 (d, *J* = 7.6, 1H Ar), 7.59 (td, *J* = 7.6, 1.1, 1H Ar), 7.45 (t, *J* = 7.6, 1H Ar), 7.19 (dd, *J* = 7.6, 0.6, 1H Ar), 6.75-6.72 (m, 2H Ar), 6.69-6.66 (m, 2H Ar), 5.42 (dd, X part of ABX system, *J* = 7.4, 3.1, 1H), 3.74 (s, 3H), 3.47 (dd, A part of ABX system, *J* = 12.8, 7.4, 1H), 3.19 (dd, B part of ABX system, J = 12.8, 3.1, 1H), 2.66 (s, 3H).

^{13}C NMR: 171.47, 167.94, 158.64, 145.55, 133.83, 130.81, 130.76, 128.82, 127.17, 125.16, 123.53, 113.73, 60.13, 55.27, 37.65, 25.73.

HPLC-MS: 10.3 min, 296 $[M+H]^+$.

GC-MS: 9.6 min, 295 (3, M), 132 (17), 121 (100), 77 (13), 43 (13).

*3-Methyl-5-(4-methylbenzyl)benzo[e][1,3]oxazepin-1(5H)-one (**14b**)*

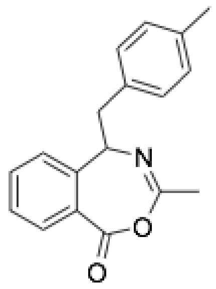

White solid, m.p.= 109.1-110.7°C

R_f = 0.52 (PE:EtOAc=4:1)

^{1}H NMR: 7.77 (d, *J* = 7.6, 1H Ar), 7.59 (td, *J* = 7.6, 1.1 Hz, 1H Ar), 7.45 (t, *J* = 7.6, 1H Ar), 7.18 (d, *J* = 7.6, 1H Ar), 6.95 (d, *J* = 8.0, 2H Ar), 6.72 (d, *J* = 8.0, 2H Ar), 5.44 (d, X part of ABX system, *J* = 7.6, 2.9, 1H), 3.50 (dd, A part of ABX system, *J* = 12.8, 7.6, 1H), 3.18 (dd, B part of ABX system, *J* = 12.8, 2.9, 1H), 2.66 (s, 3H), 2.26 (s, 3H).

^{13}C NMR: 171.46, 167.95, 145.57, 136.62, 133.80, 132.09, 130.79, 129.65, 129.03, 128.80, 125.12, 123.56, 60.06, 38.11, 25.74, 21.19.

HPLC-MS: 11.8 min, 280 $[M+H]^+$.

GC-MS: 8.9 min, 279 (9, M), 132 (100), 105 (48), 77 (13), 43 (14).

Compound 4a

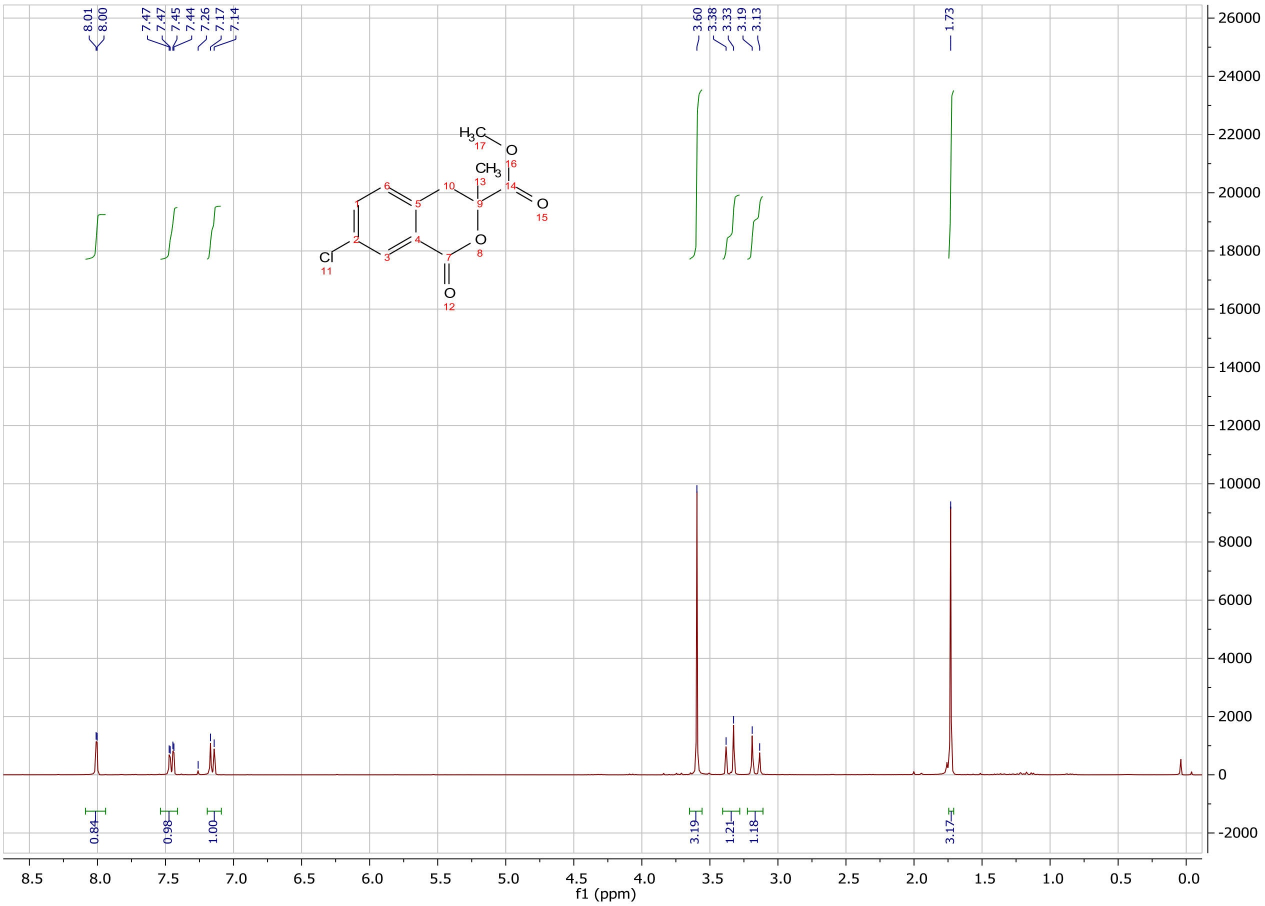

8.01
8.00
7.47
7.47
7.45
7.44
7.26
7.17
7.14
3.60
3.38
3.33
3.19
3.13
1.73
0.84
0.98
1.00
3.19
1.21
1.18
3.17
f1 (ppm)

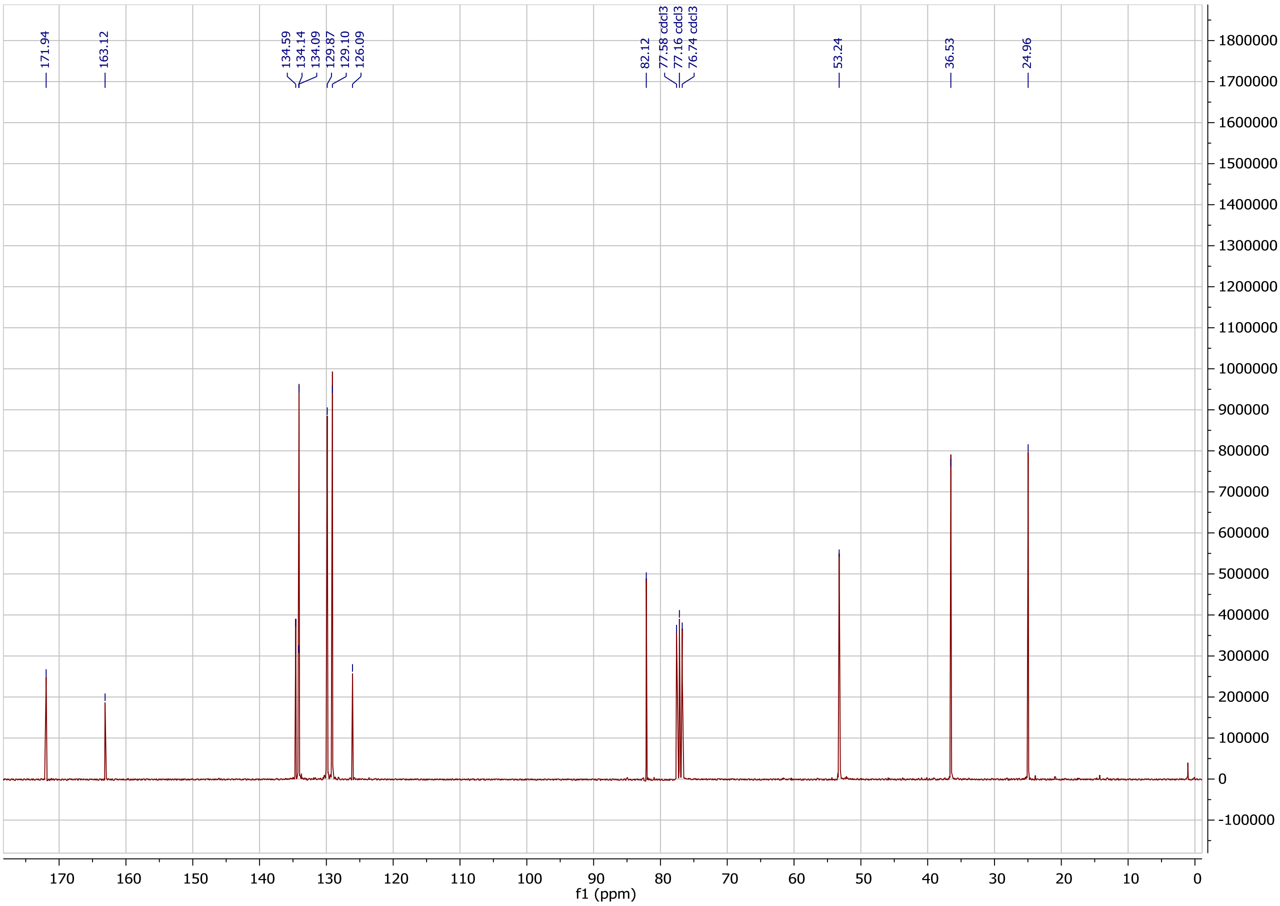

171.94
163.12
134.59
134.14
134.09
129.87
129.10
126.09
82.12
77.58 cdcl3
77.16 cdcl3
76.74 cdcl3
53.24
36.53
24.96
f1 (ppm)

Compound 4b

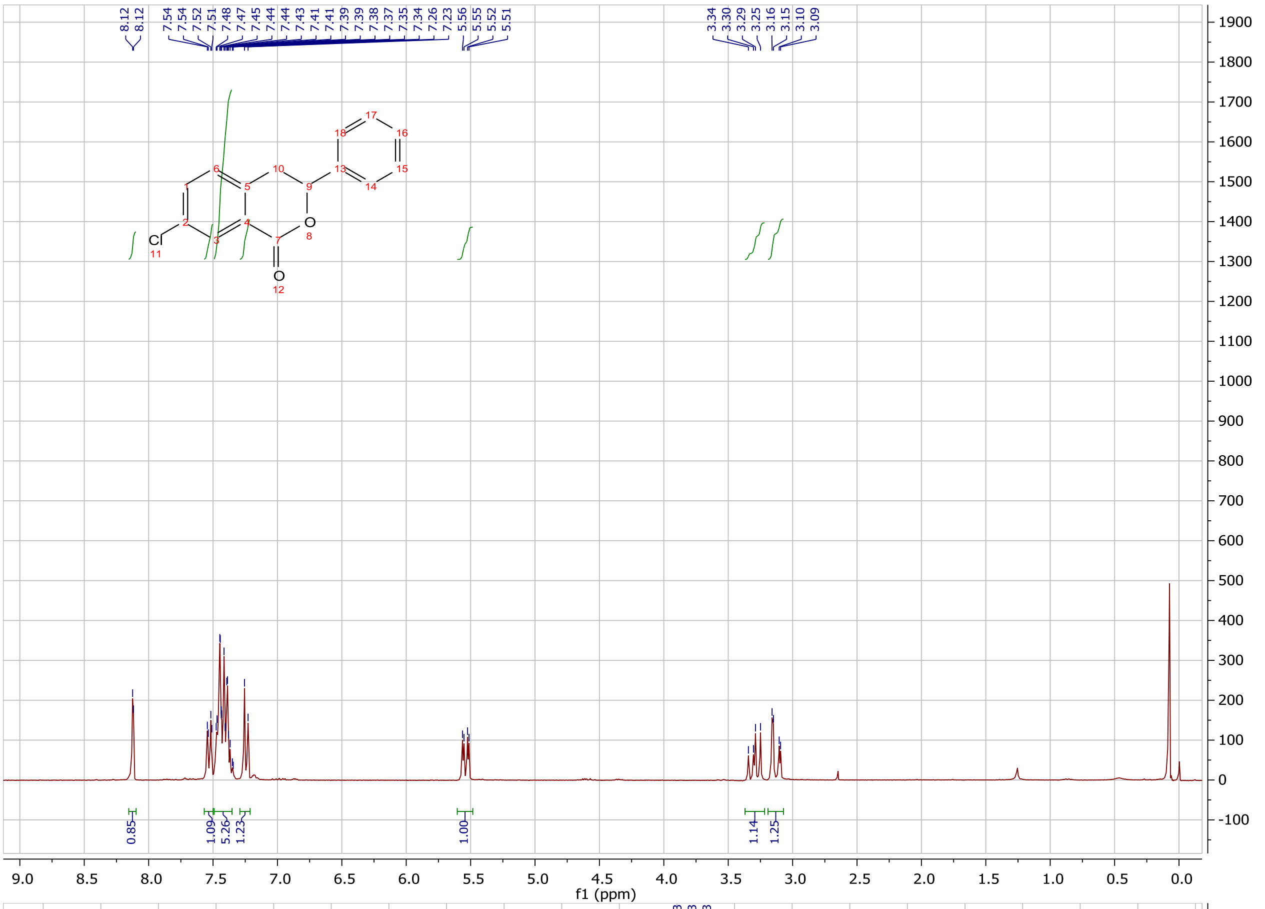
8.12
8.12
7.54
7.54
7.52
7.51
7.48
7.47
7.45
7.44
7.44
7.43
7.41
7.41
7.39
7.39
7.38
7.37
7.35
7.34
7.26
7.23
5.56
5.55
5.52
5.51
3.34
3.30
3.29
3.25
3.16
3.15
3.10
3.09
Cl
O
O
0.85
1.09
5.26
1.23
1.00
1.14
1.25
f1 (ppm)

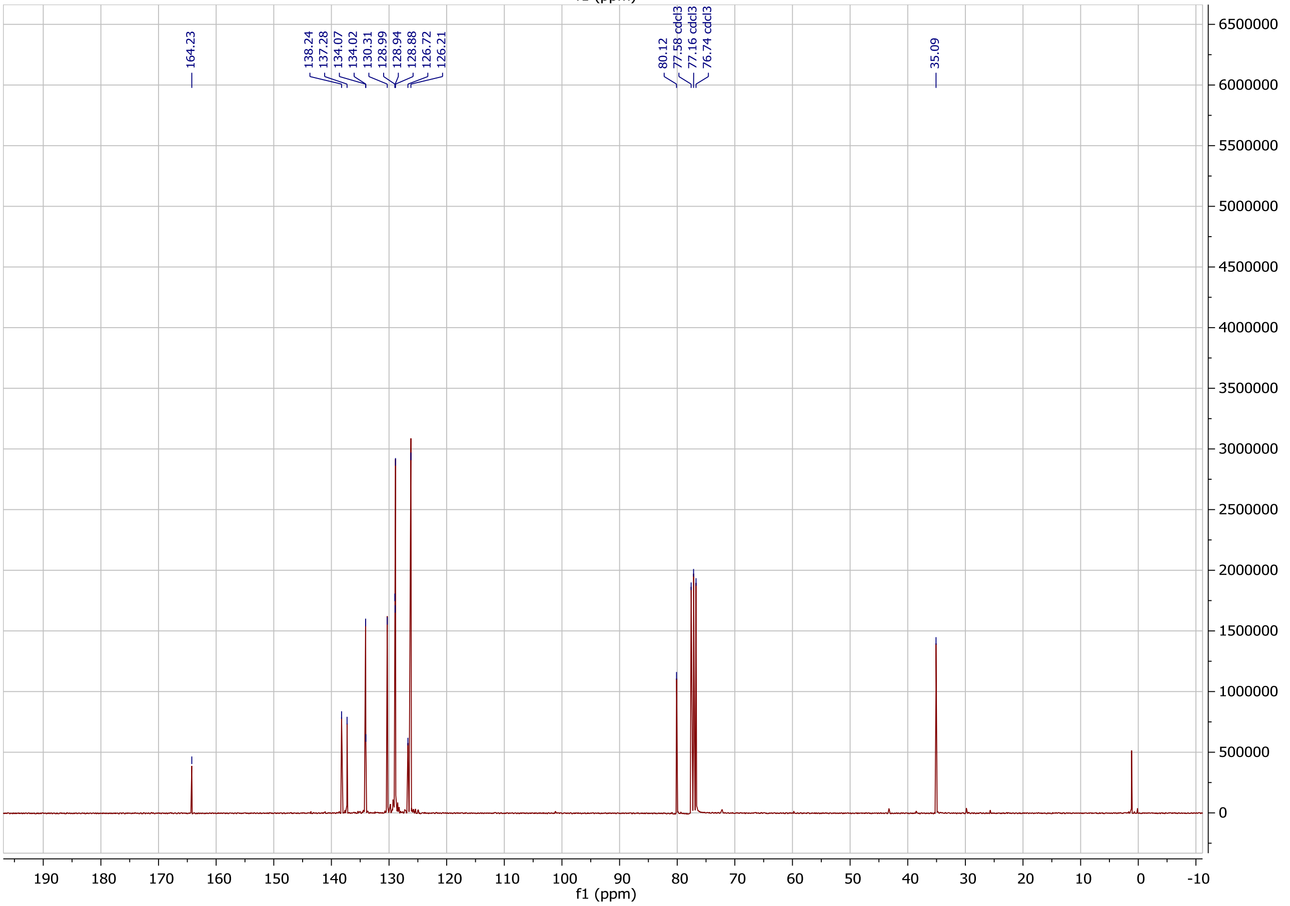
164.23
138.24
137.28
134.07
134.02
130.31
128.99
128.94
128.88
126.72
126.21
80.12
77.58 cdcl3
77.16 cdcl3
76.74 cdcl3
35.09
f1 (ppm)

Compound 4c

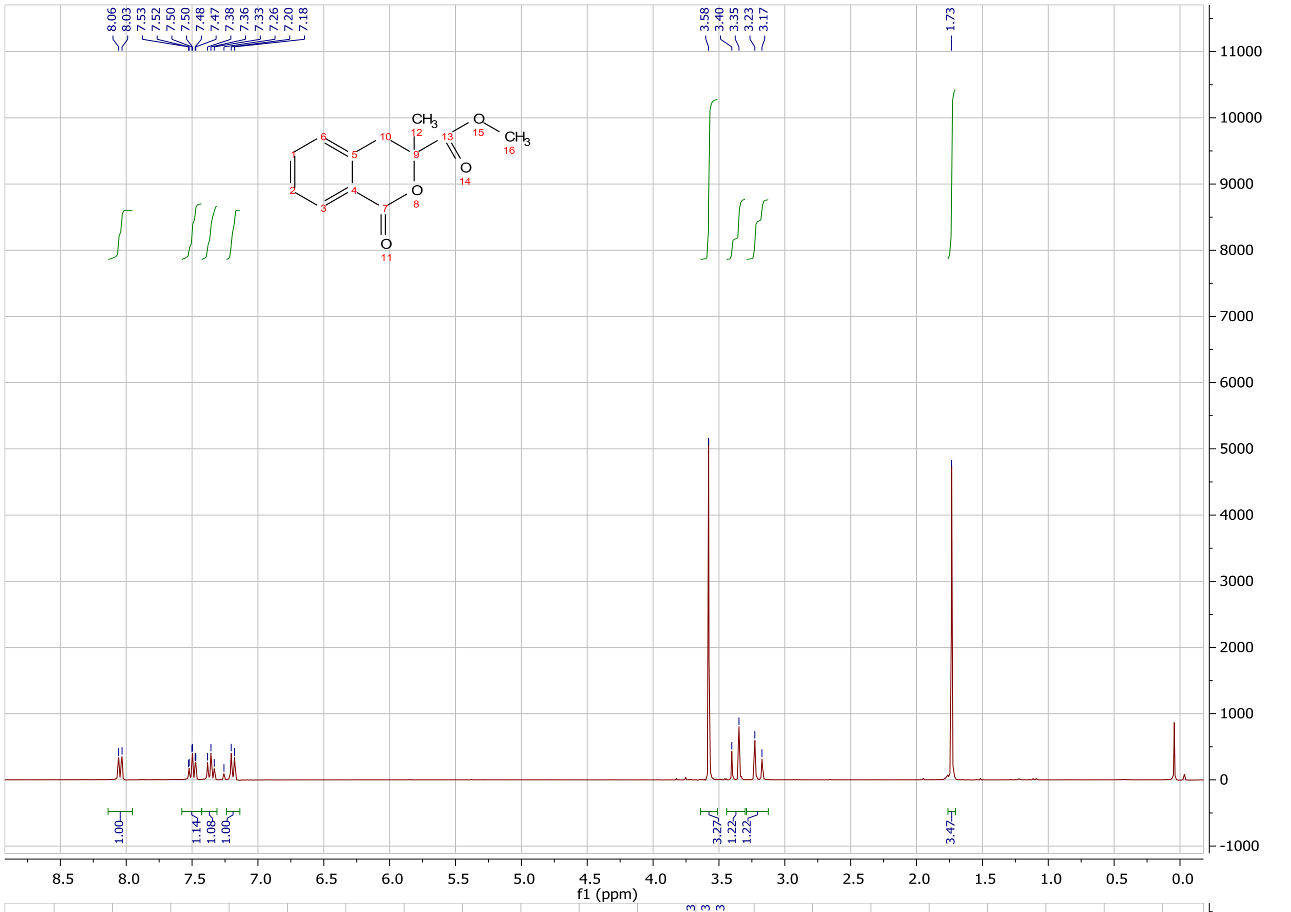
8.06
8.03
7.53
7.52
7.50
7.50
7.48
7.47
7.38
7.36
7.33
7.26
7.20
7.18
3.58
3.40
3.35
3.23
3.17
1.73
1.00
1.14
1.08
1.00
3.27
1.22
1.22
3.47
f1 (ppm)

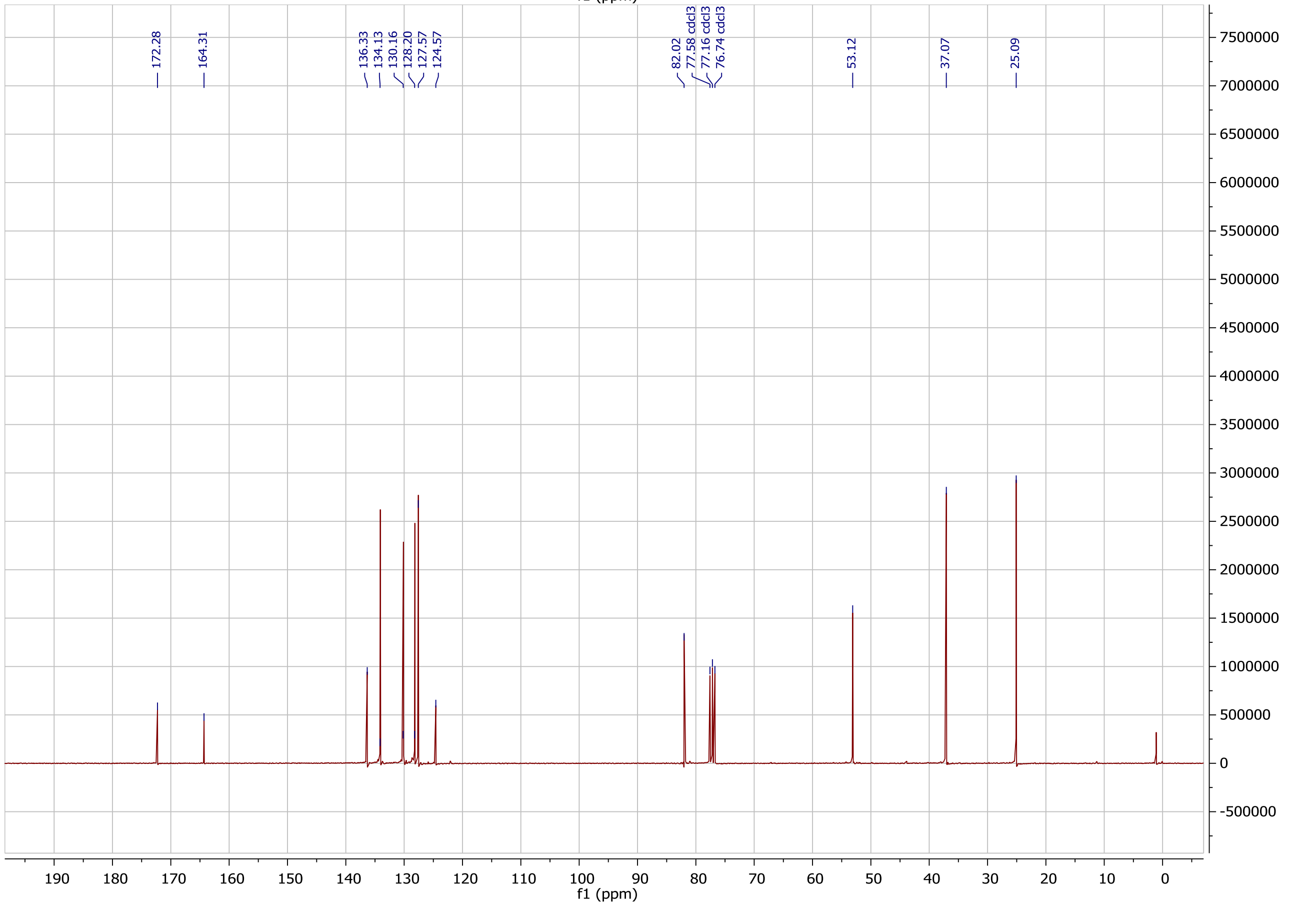
172.28
164.31
136.33
134.13
130.16
128.20
127.57
124.57
82.02
77.58 cdcl3
77.16 cdcl3
76.74 cdcl3
53.12
37.07
25.09
f1 (ppm)

Compound 4d

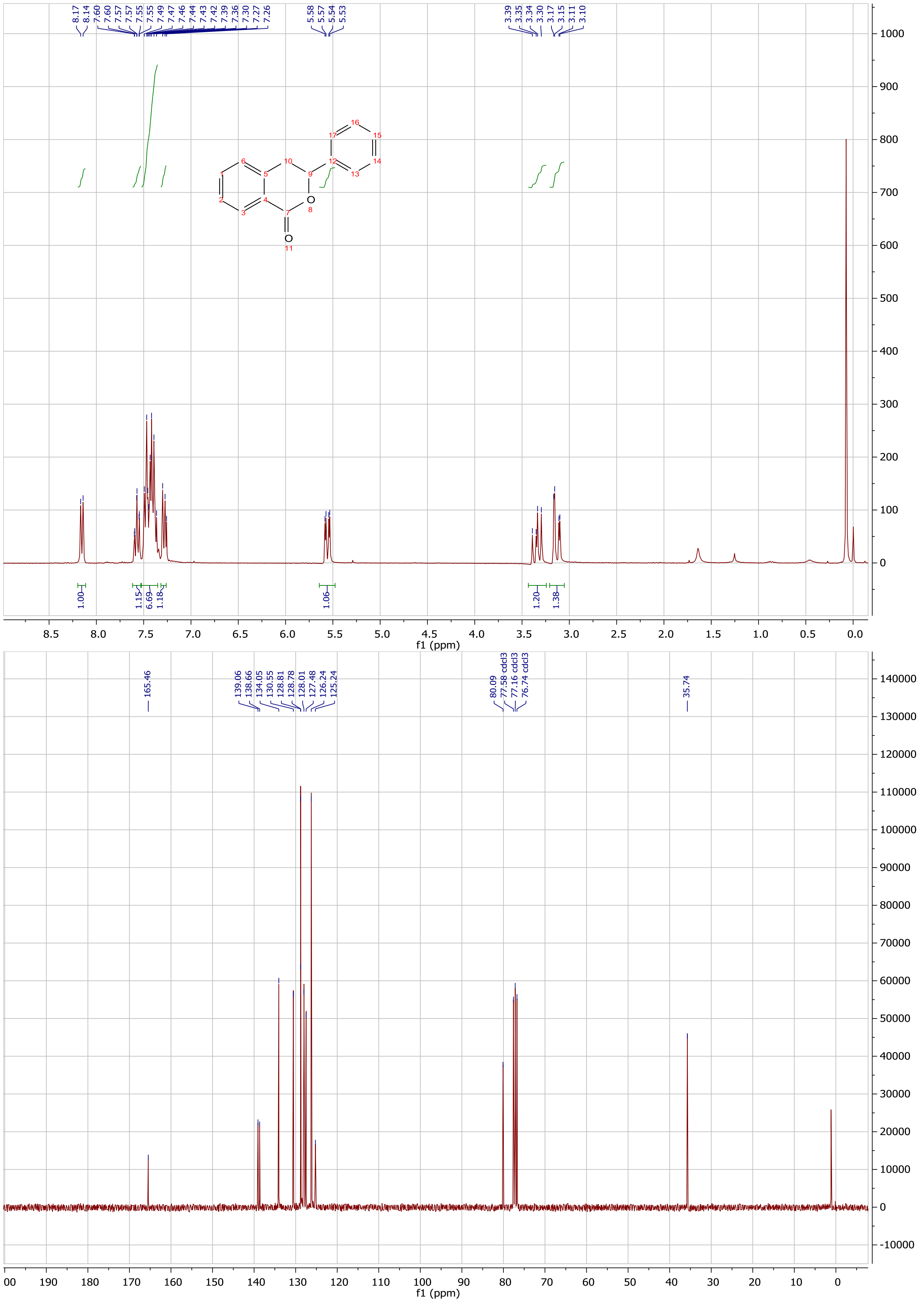
8.17
8.14
7.60
7.60
7.57
7.57
7.55
7.55
7.49
7.47
7.46
7.44
7.43
7.42
7.39
7.36
7.30
7.27
7.26
5.58
5.57
5.54
5.53
3.39
3.35
3.34
3.30
3.17
3.15
3.11
3.10
1.00
1.15
6.69
1.18
1.06
1.20
1.38
f1 (ppm)
165.46
139.06
138.66
134.05
130.55
128.81
128.78
128.01
127.48
126.24
125.24
80.09
77.58 cdcl3
77.16 cdcl3
76.74 cdcl3
35.74
f1 (ppm)

Compound 4e

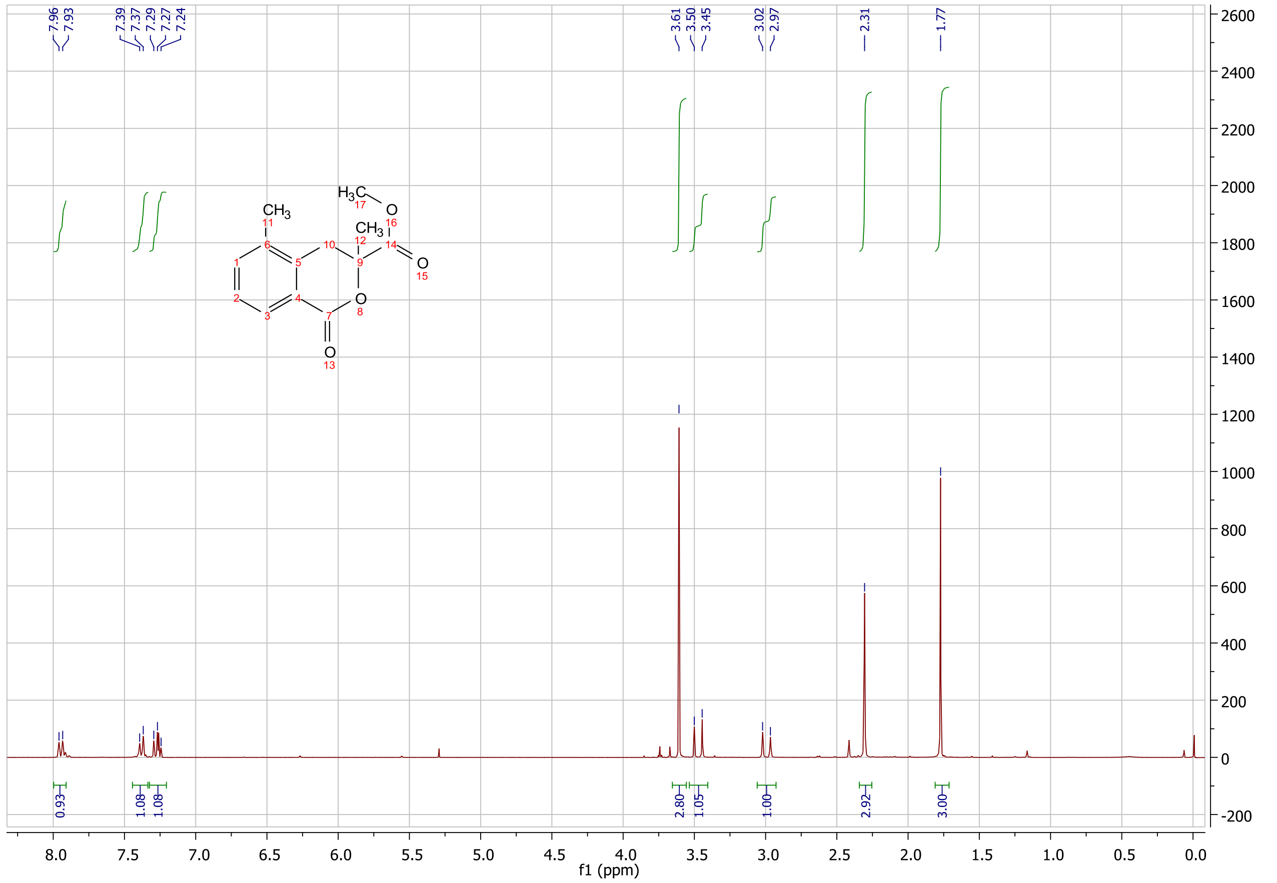

7.96
7.93
7.39
7.37
7.29
7.27
7.24
3.61
3.50
3.45
3.02
2.97
2.31
1.77
0.93
1.08
1.08
2.80
1.05
1.00
2.92
3.00
f1 (ppm)

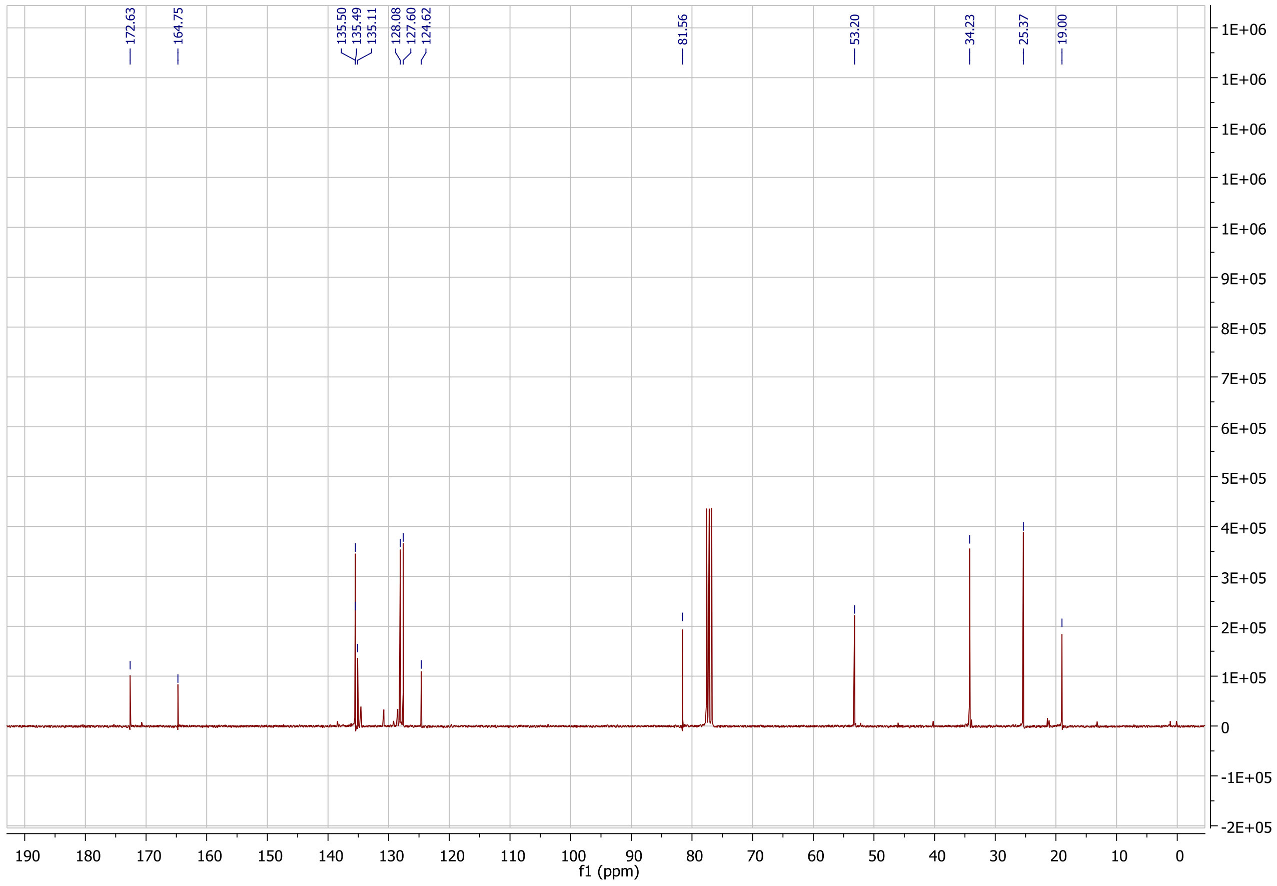

172.63
164.75
135.50
135.49
135.11
128.08
127.60
124.62
81.56
53.20
34.23
25.37
19.00
f1 (ppm)

Compound 4f

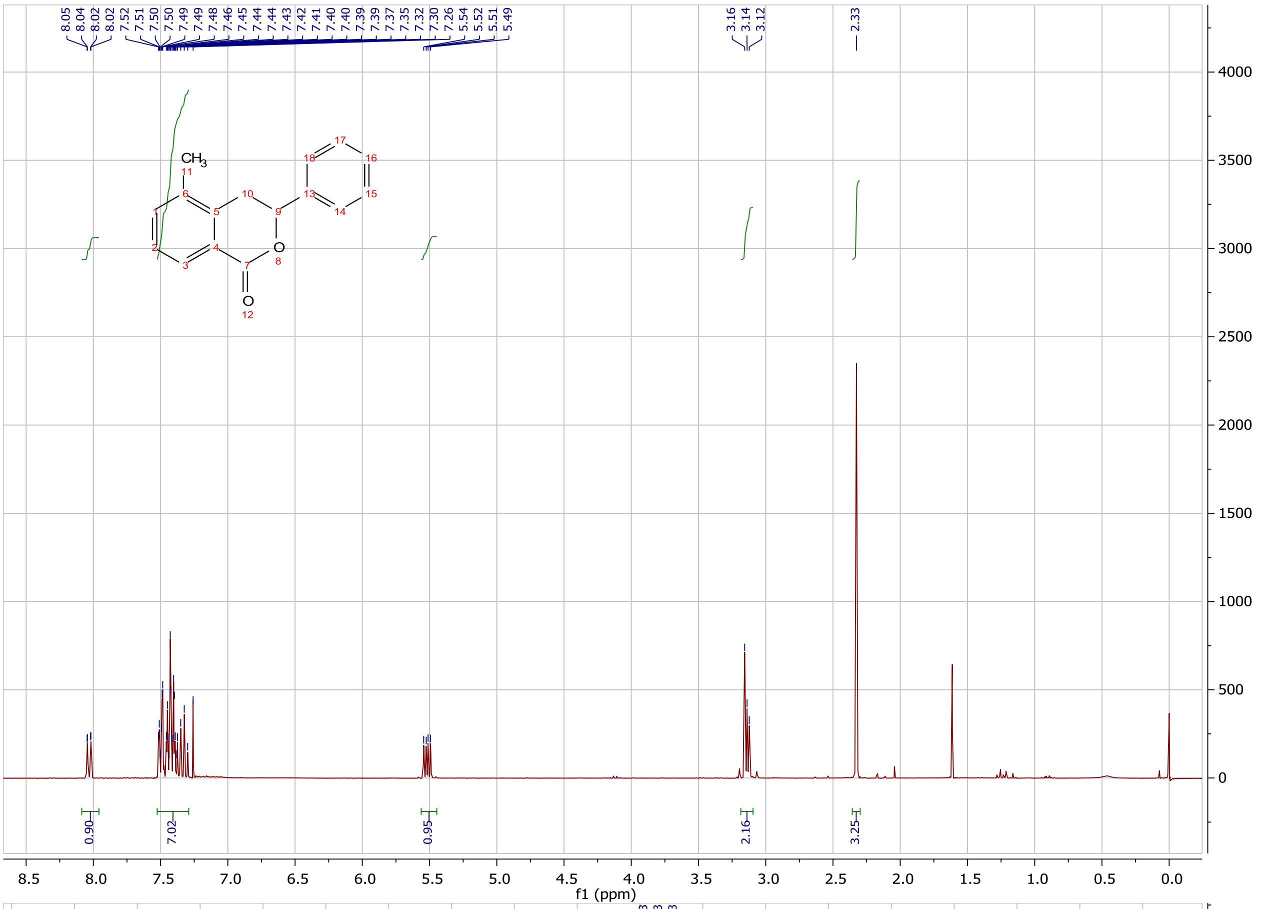
8.05 8.04 8.02 8.02 7.52 7.51 7.50 7.50 7.49 7.48 7.46 7.45 7.44 7.44 7.43 7.42 7.41 7.40 7.40 7.39 7.39 7.37 7.35 7.32 7.30 7.26 5.54 5.52 5.51 5.49
3.16 3.14 3.12
2.33
CH3
O
O
0.90
7.02
0.95
2.16
3.25
f1 (ppm)

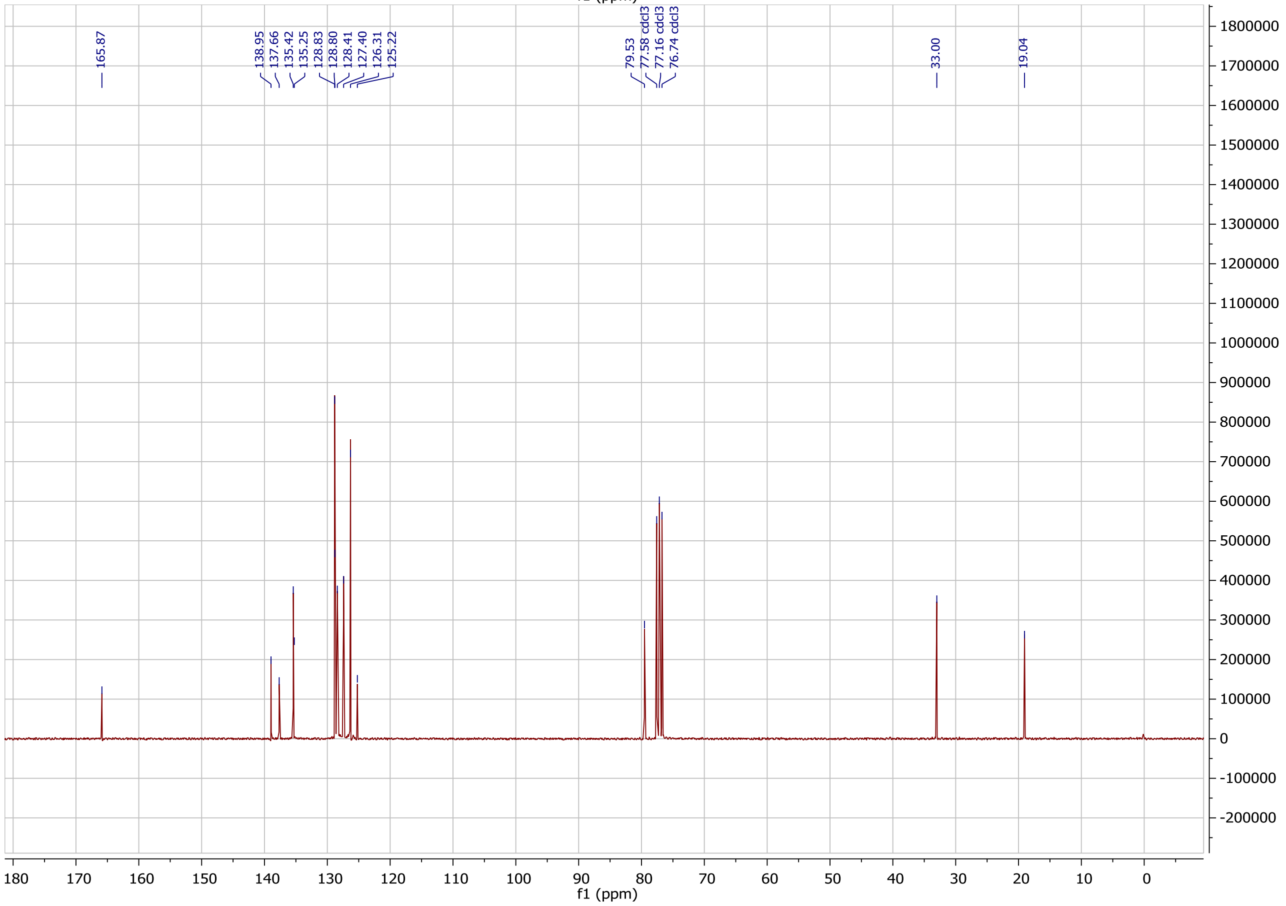
165.87
138.95 137.66 135.42 135.25 128.83 128.80 128.41 127.40 126.31 125.22
79.53 77.58 cdcl3 77.16 cdcl3 76.74 cdcl3
33.00
19.04
f1 (ppm)

Compound 4g

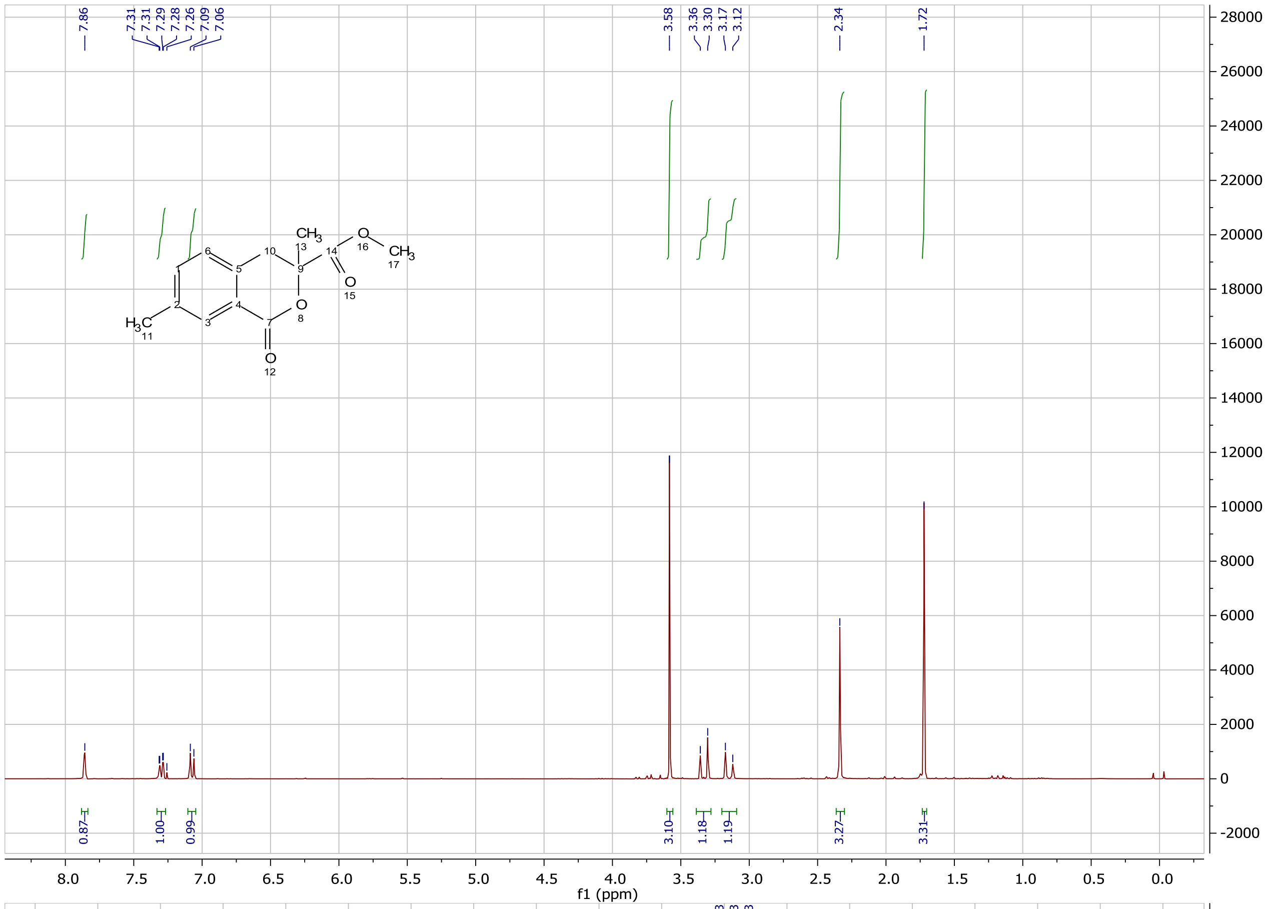

7.86
7.31
7.31
7.29
7.28
7.26
7.09
7.06
3.58
3.36
3.30
3.17
3.12
2.34
1.72
0.87
1.00
0.99
3.10
1.18
1.19
3.27
3.31
f1 (ppm)

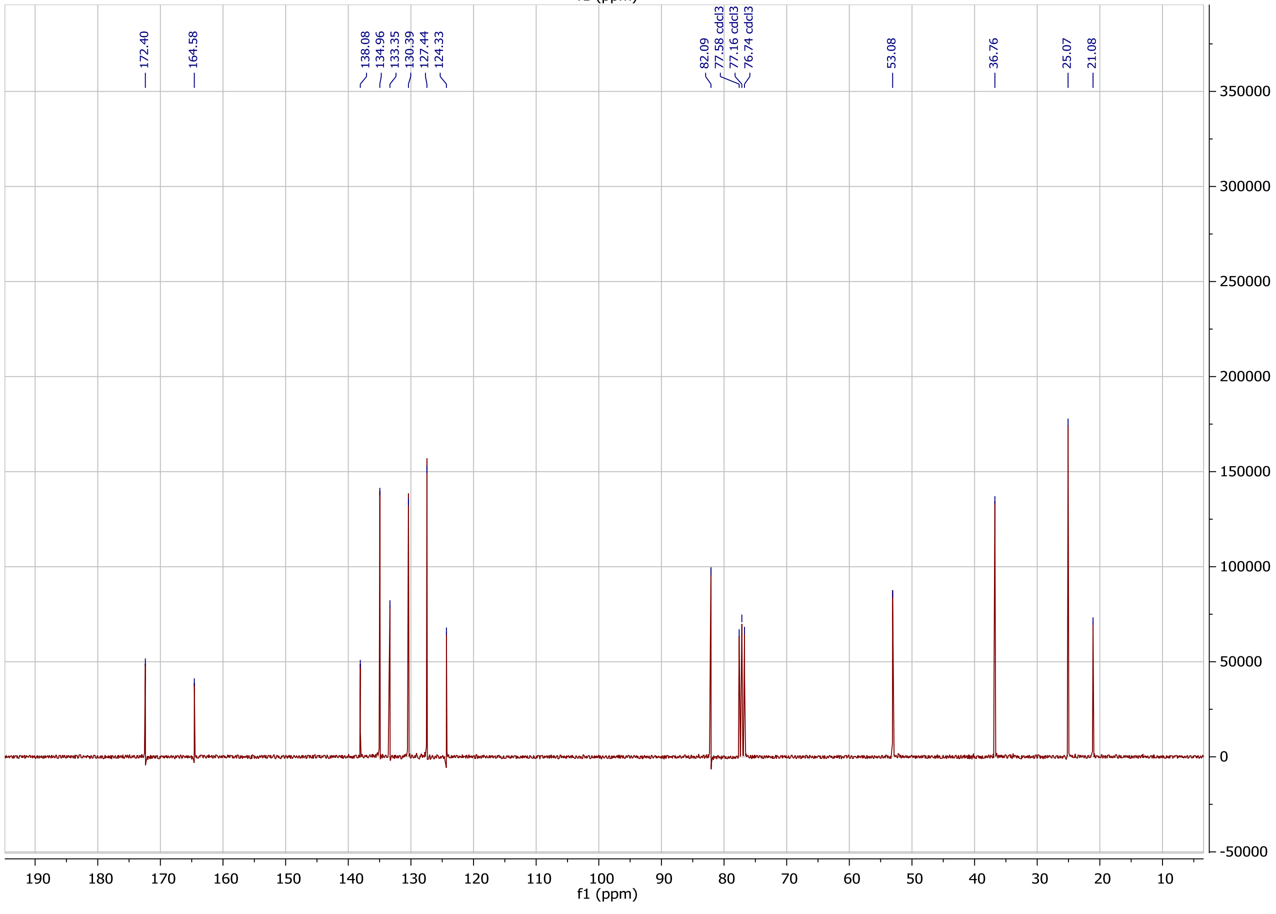

172.40
164.58
138.08
134.96
133.35
130.39
127.44
124.33
82.09
77.58 cdcl3
77.16 cdcl3
76.74 cdcl3
53.08
36.76
25.07
21.08
f1 (ppm)

Compound 4h

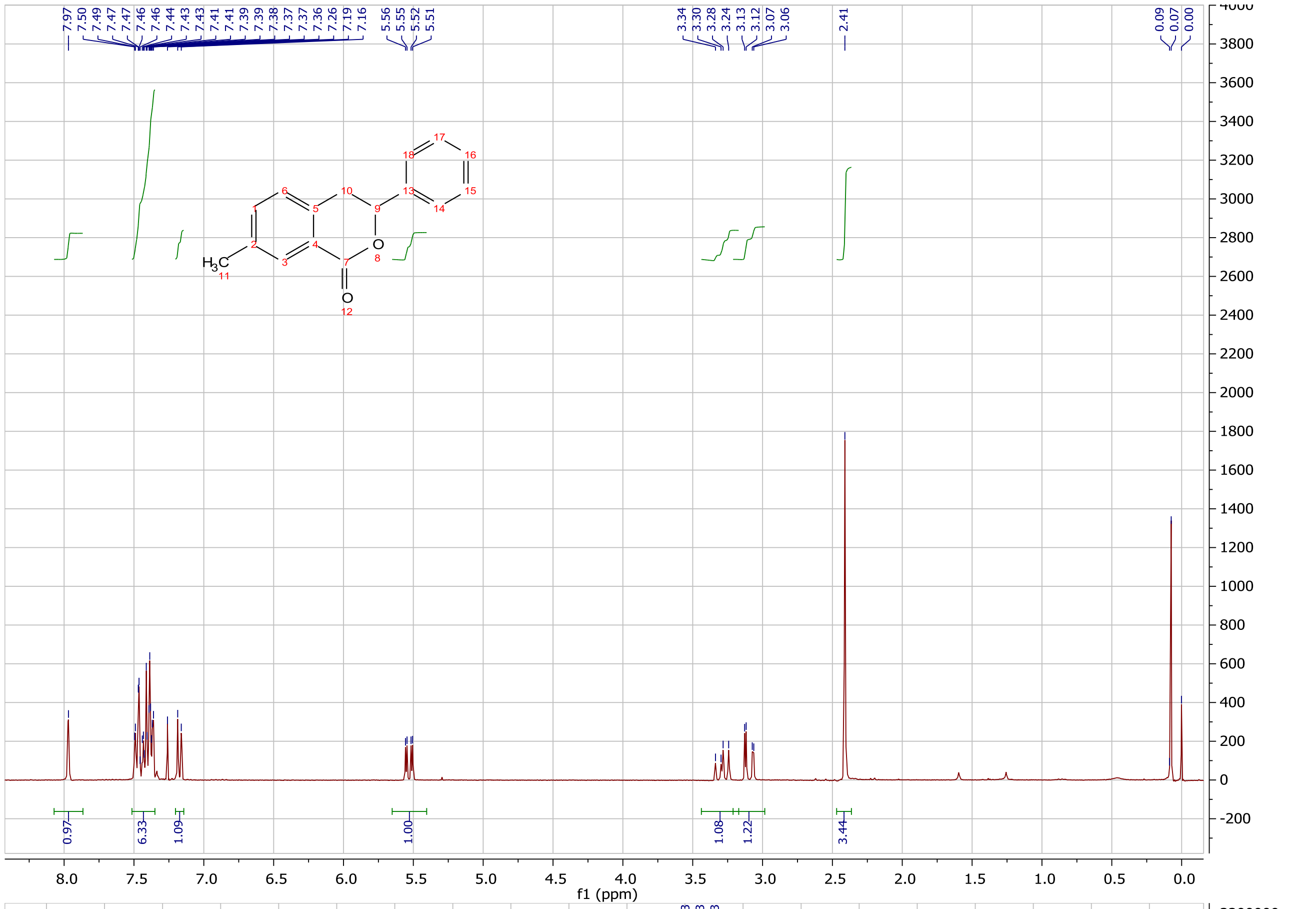
7.97
7.50
7.49
7.47
7.47
7.46
7.46
7.44
7.43
7.43
7.41
7.41
7.39
7.39
7.38
7.37
7.37
7.36
7.26
7.19
7.16
5.56
5.55
5.52
5.51
3.34
3.30
3.28
3.24
3.13
3.12
3.07
3.06
2.41
0.09
0.07
0.00
0.97
6.33
1.09
1.00
1.08
1.22
3.44
f1 (ppm)

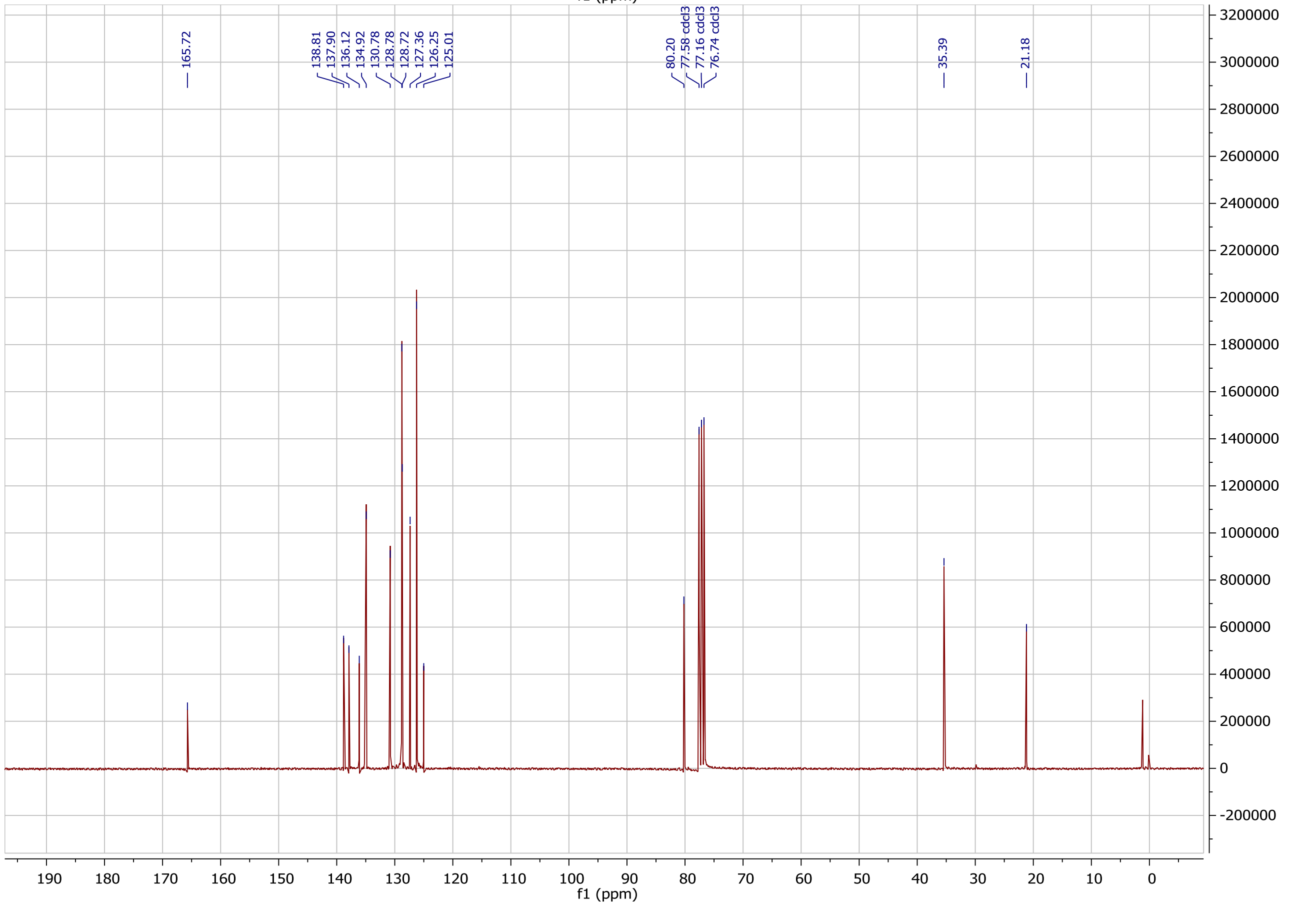
165.72
138.81
137.90
136.12
134.92
130.78
128.78
128.72
127.36
126.25
125.01
80.20
77.58 cdcl3
77.16 cdcl3
76.74 cdcl3
35.39
21.18
f1 (ppm)

Compound 4i

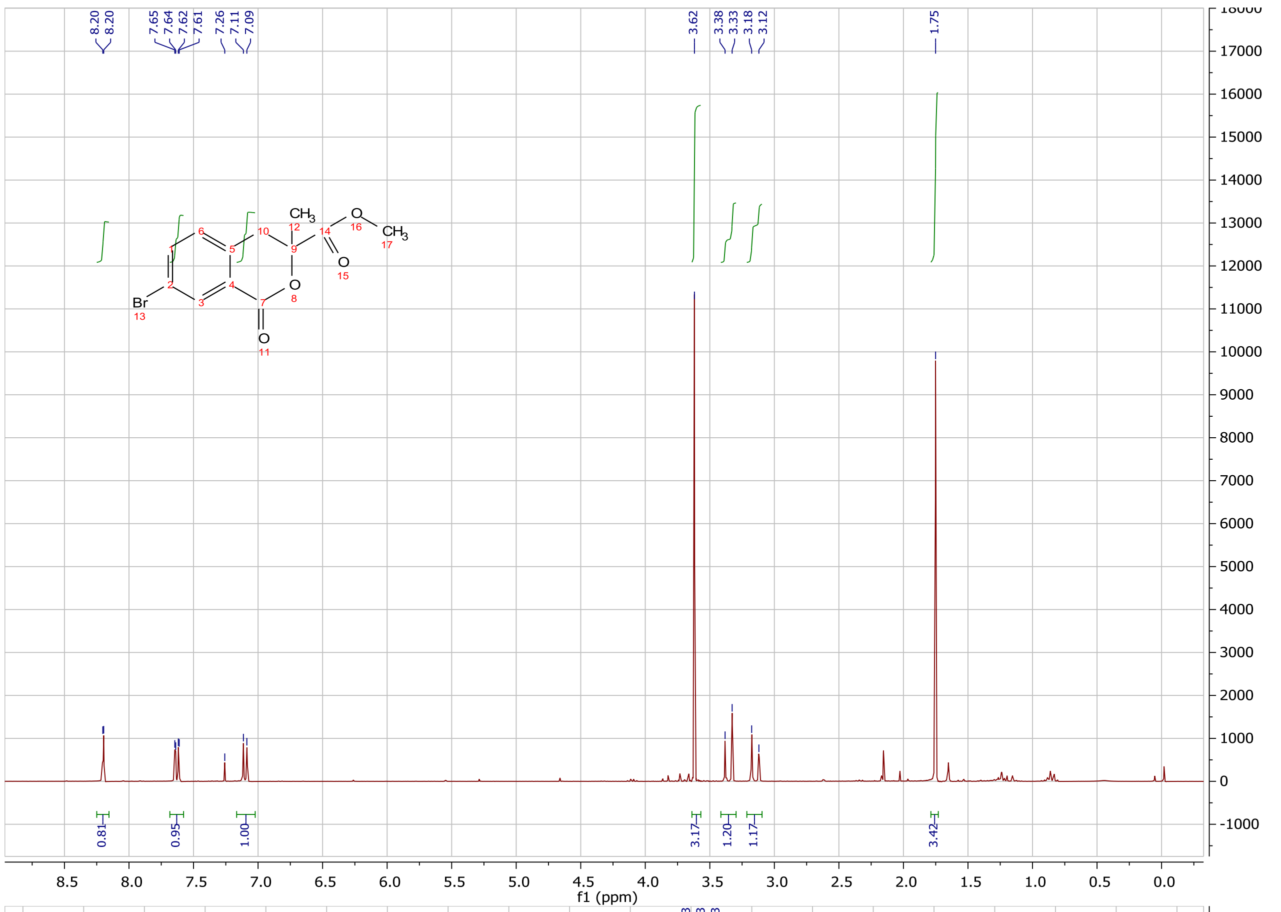
8.20
8.20
7.65
7.64
7.62
7.61
7.26
7.11
7.09
3.62
3.38
3.33
3.18
3.12
1.75
0.81
0.95
1.00
3.17
1.20
1.17
3.42
f1 (ppm)

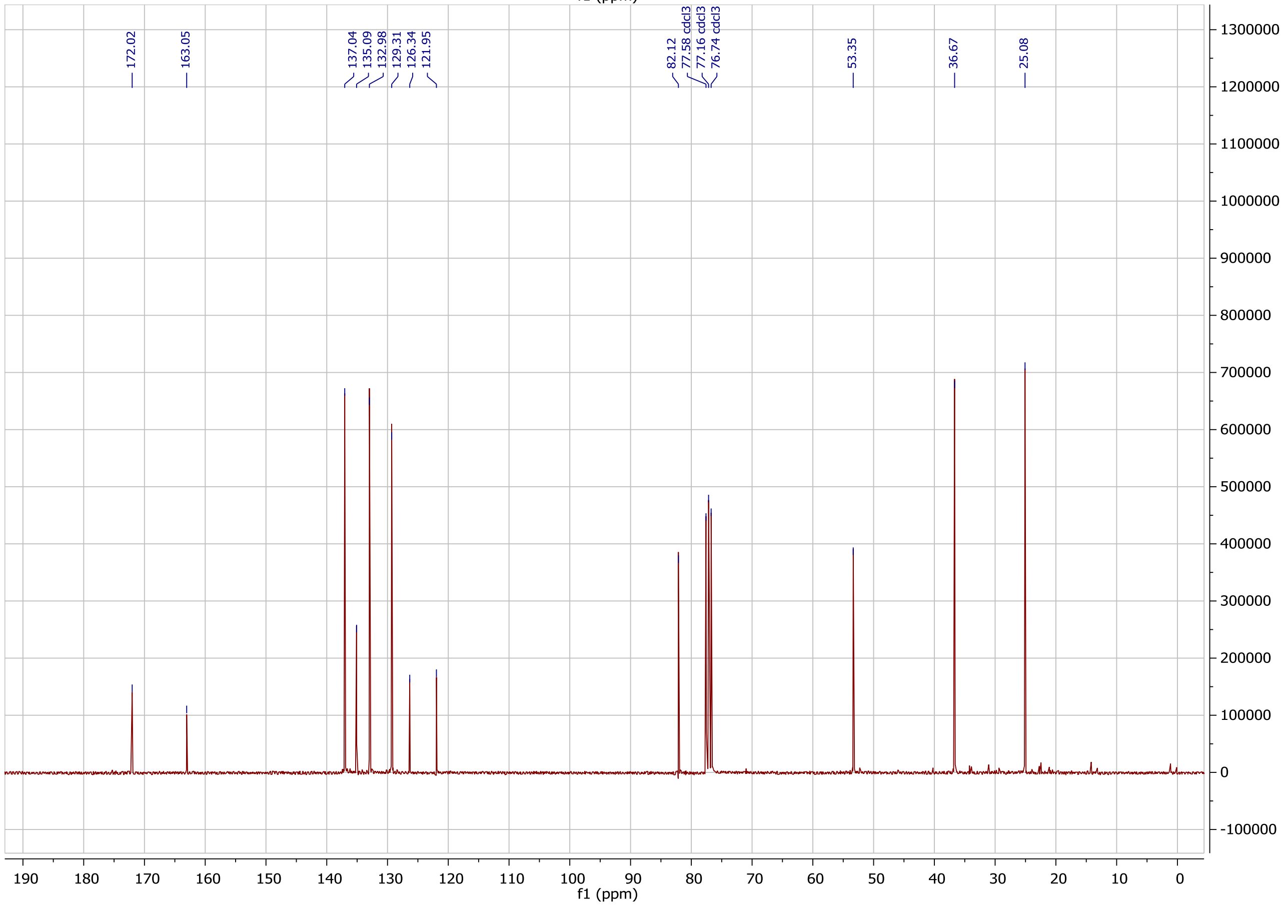
172.02
163.05
137.04
135.09
132.98
129.31
126.34
121.95
82.12
77.58 cdcl3
77.16 cdcl3
76.74 cdcl3
53.35
36.67
25.08
f1 (ppm)

Compound 4j

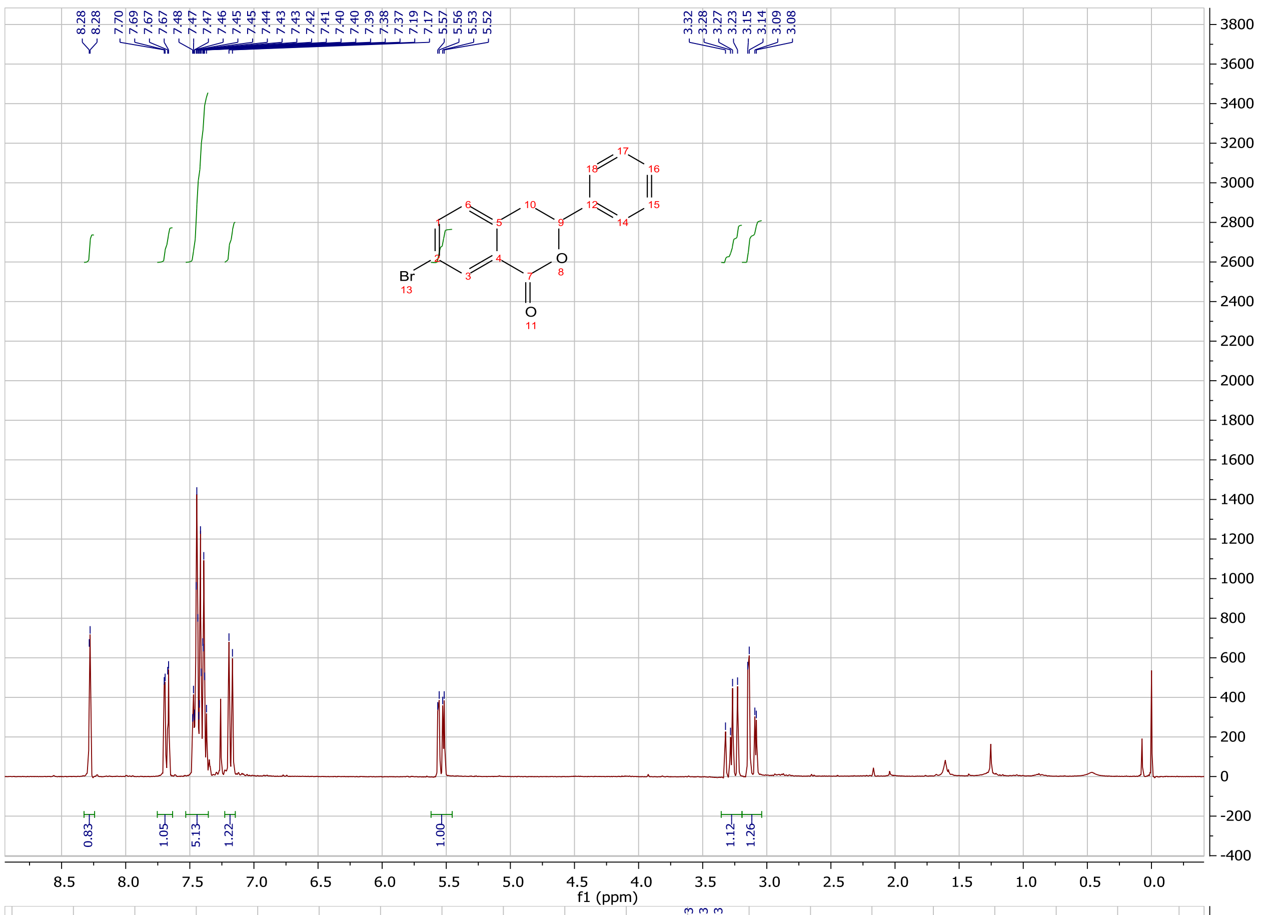
8.28
8.28
7.70
7.69
7.67
7.67
7.48
7.47
7.47
7.46
7.45
7.45
7.44
7.43
7.43
7.42
7.41
7.40
7.40
7.39
7.38
7.37
7.19
7.17
5.57
5.56
5.53
5.52
3.32
3.28
3.27
3.23
3.15
3.14
3.09
3.08
Br
O
O
0.83
1.05
5.13
1.22
1.00
1.12
1.26
f1 (ppm)

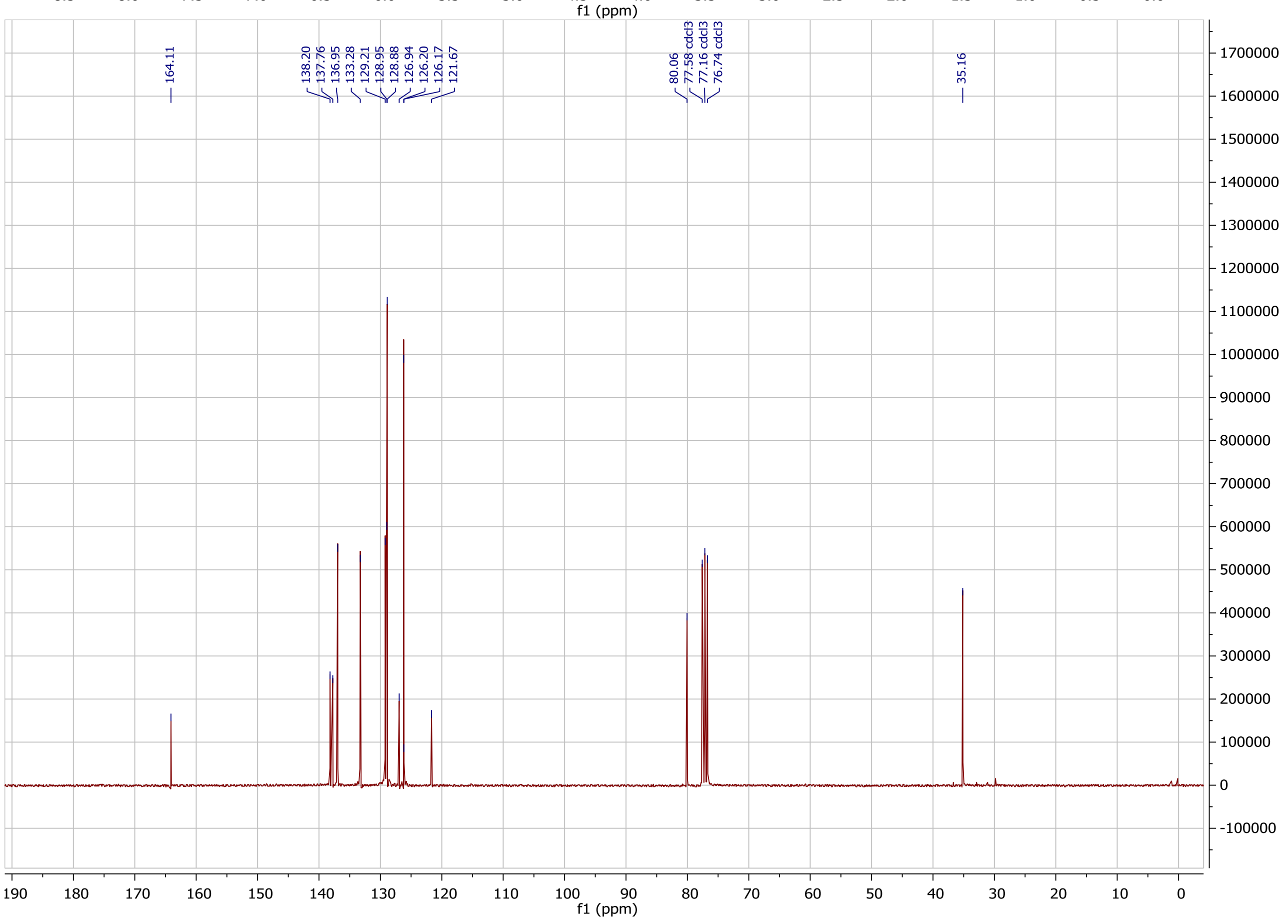
164.11
138.20
137.76
136.95
133.28
129.21
128.95
128.88
126.94
126.20
126.17
121.67
80.06
77.58 cdcl3
77.16 cdcl3
76.74 cdcl3
35.16
f1 (ppm)

Compound 4k

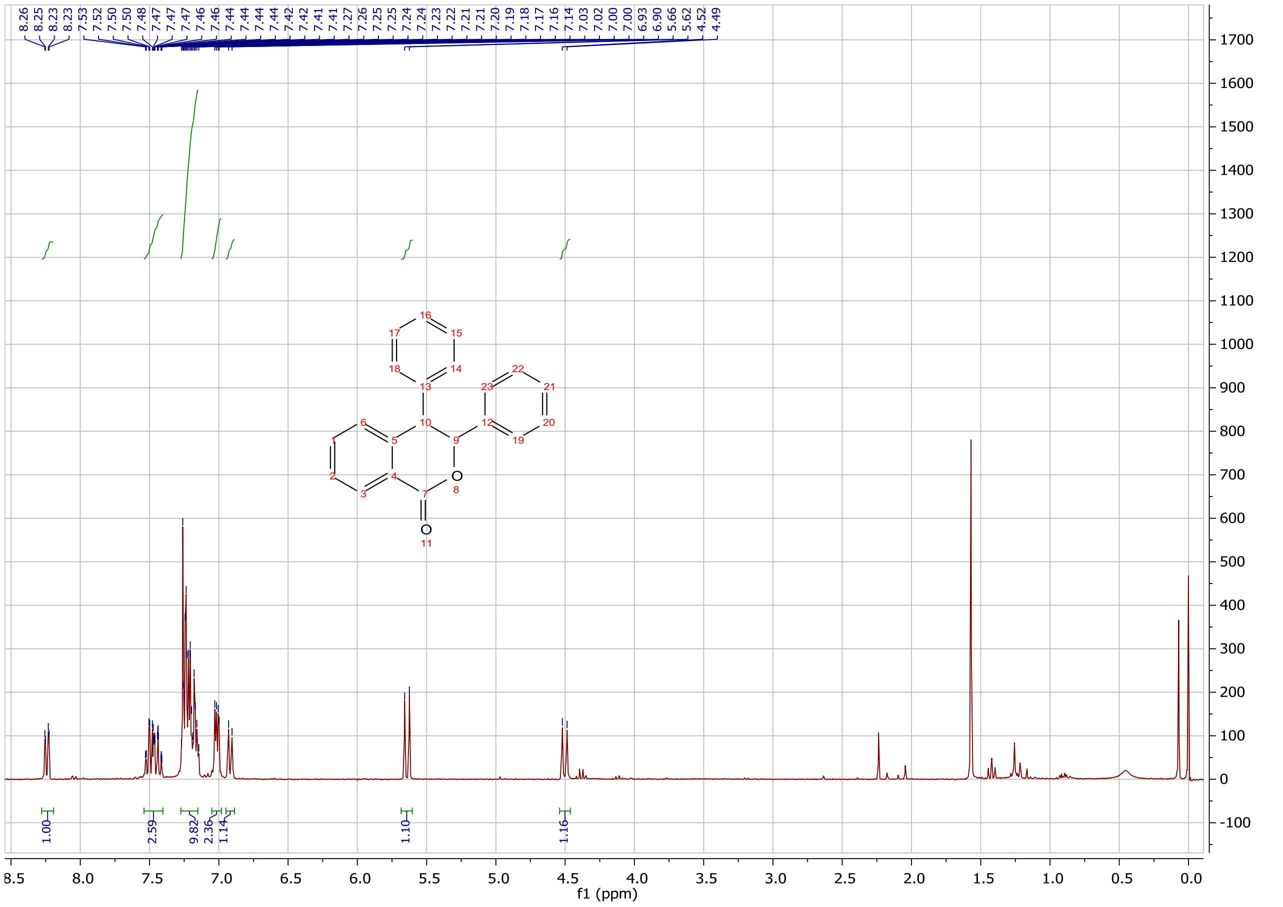

8.26
8.25
8.23
8.23
7.53
7.52
7.50
7.50
7.48
7.47
7.47
7.47
7.46
7.44
7.44
7.44
7.44
7.42
7.42
7.41
7.41
7.27
7.26
7.25
7.25
7.24
7.24
7.23
7.22
7.21
7.21
7.20
7.19
7.18
7.17
7.16
7.14
7.03
7.02
7.00
7.00
6.93
6.90
5.66
5.62
4.52
4.49
1.00
2.59
9.82
2.36
1.14
1.10
1.16
f1 (ppm)

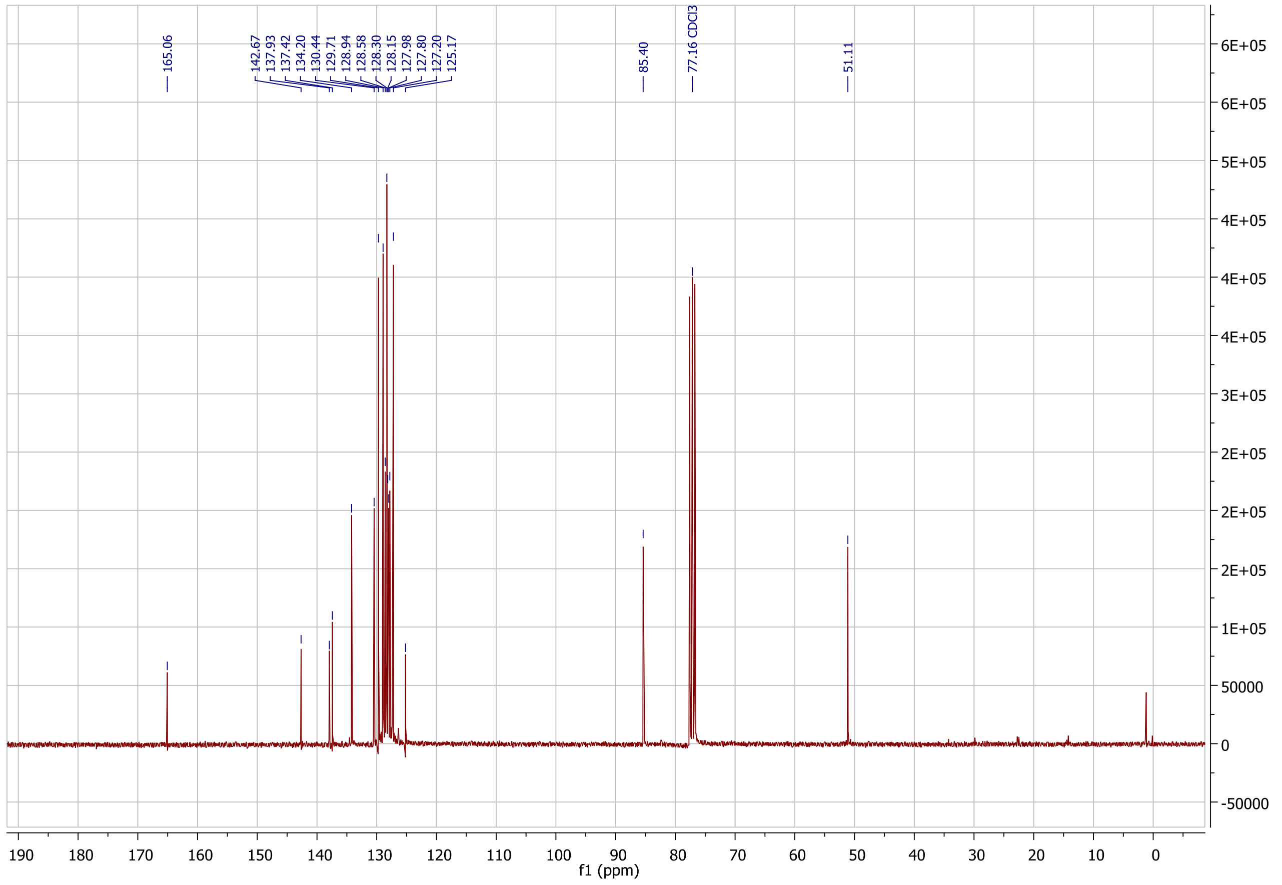

165.06
142.67
137.93
137.42
134.20
130.44
129.71
128.94
128.58
128.30
128.15
127.98
127.80
127.20
125.17
85.40
77.16 CDCl3
51.11
f1 (ppm)

Compound 5

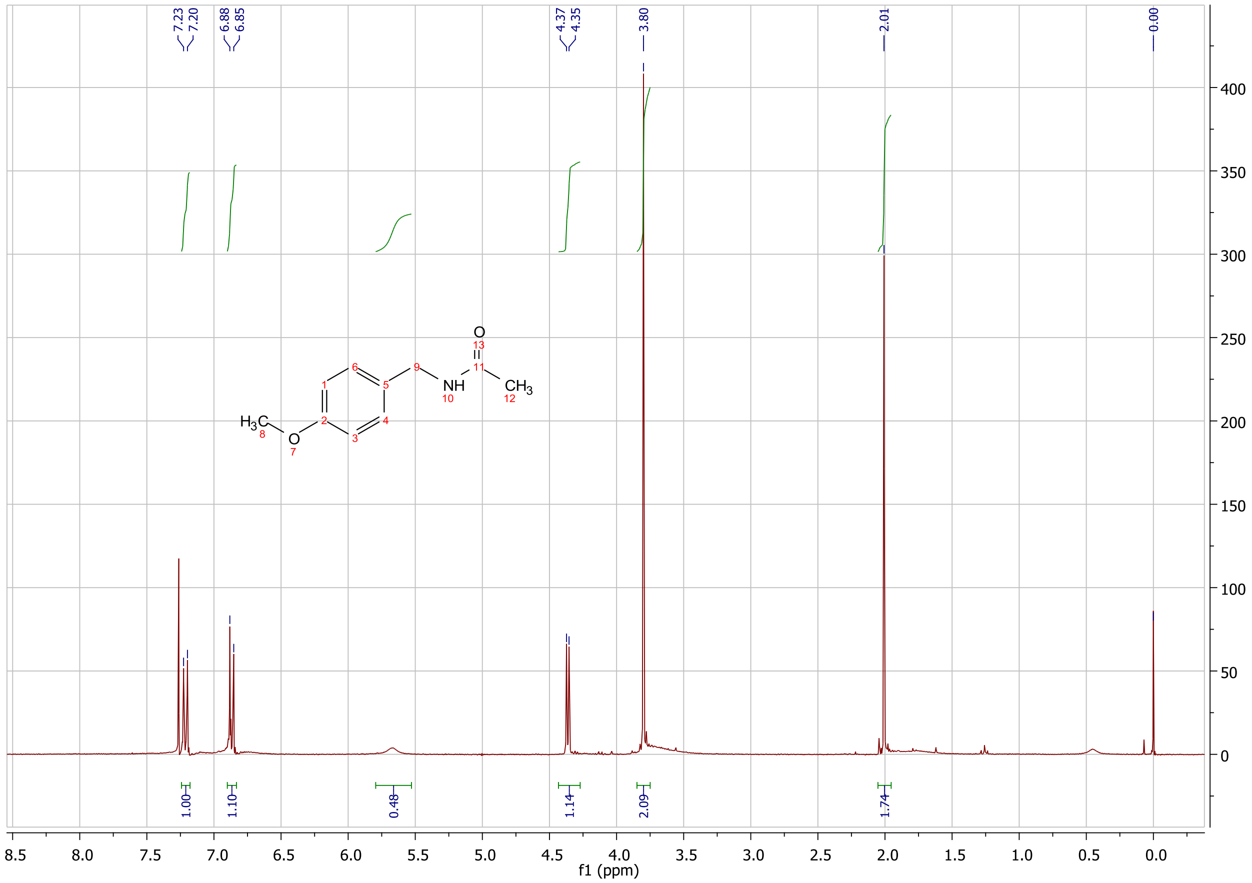
7.23
7.20
6.88
6.85
4.37
4.35
3.80
2.01
0.00
H3C
O
NH
CH3
O
1.00
1.10
0.48
1.14
2.09
1.74
f1 (ppm)

Compound 9

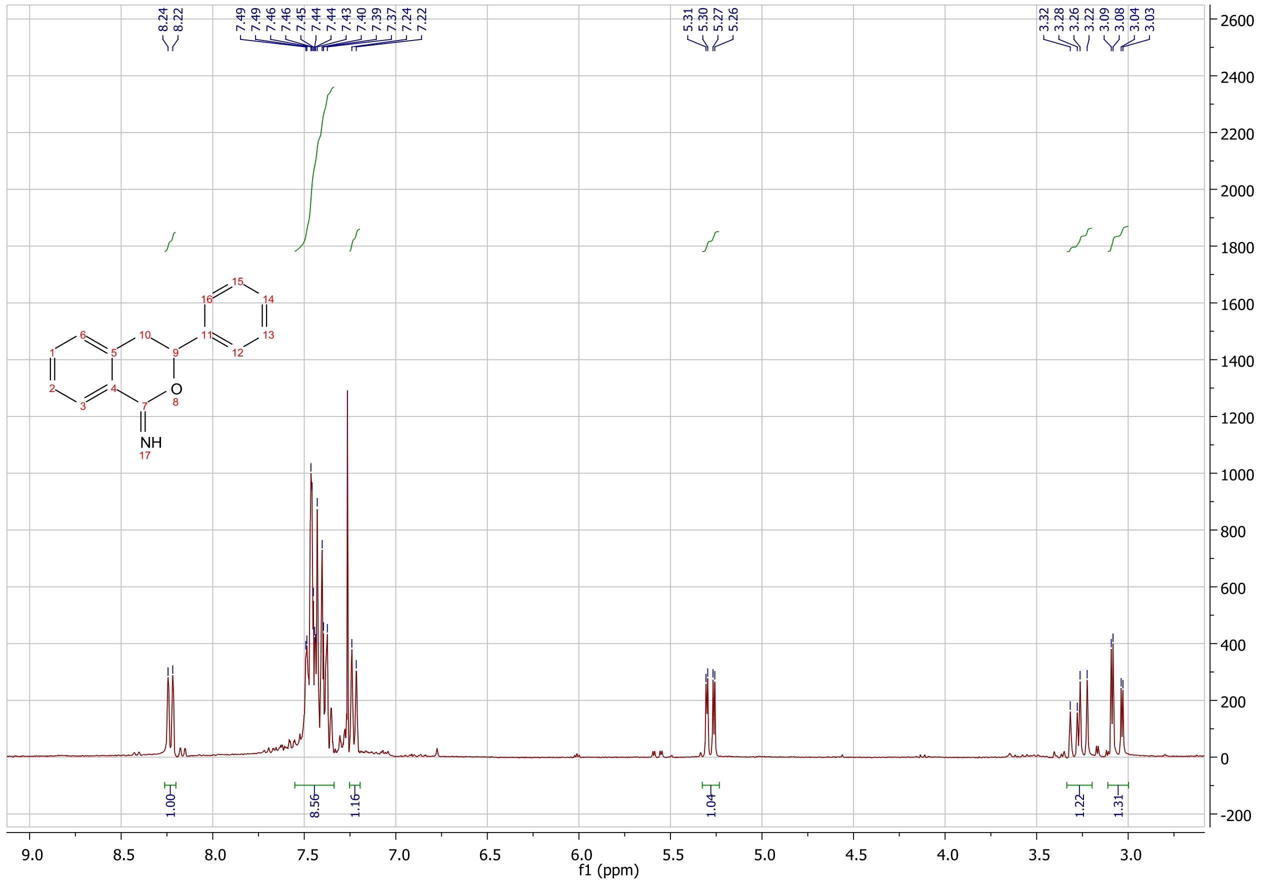

8.24
8.22
7.49
7.49
7.46
7.45
7.44
7.44
7.43
7.40
7.39
7.37
7.24
7.22
5.31
5.30
5.27
5.26
3.32
3.28
3.26
3.22
3.09
3.08
3.04
3.03
NH
O
1.00
8.56
1.16
1.04
1.22
1.31
f1 (ppm)

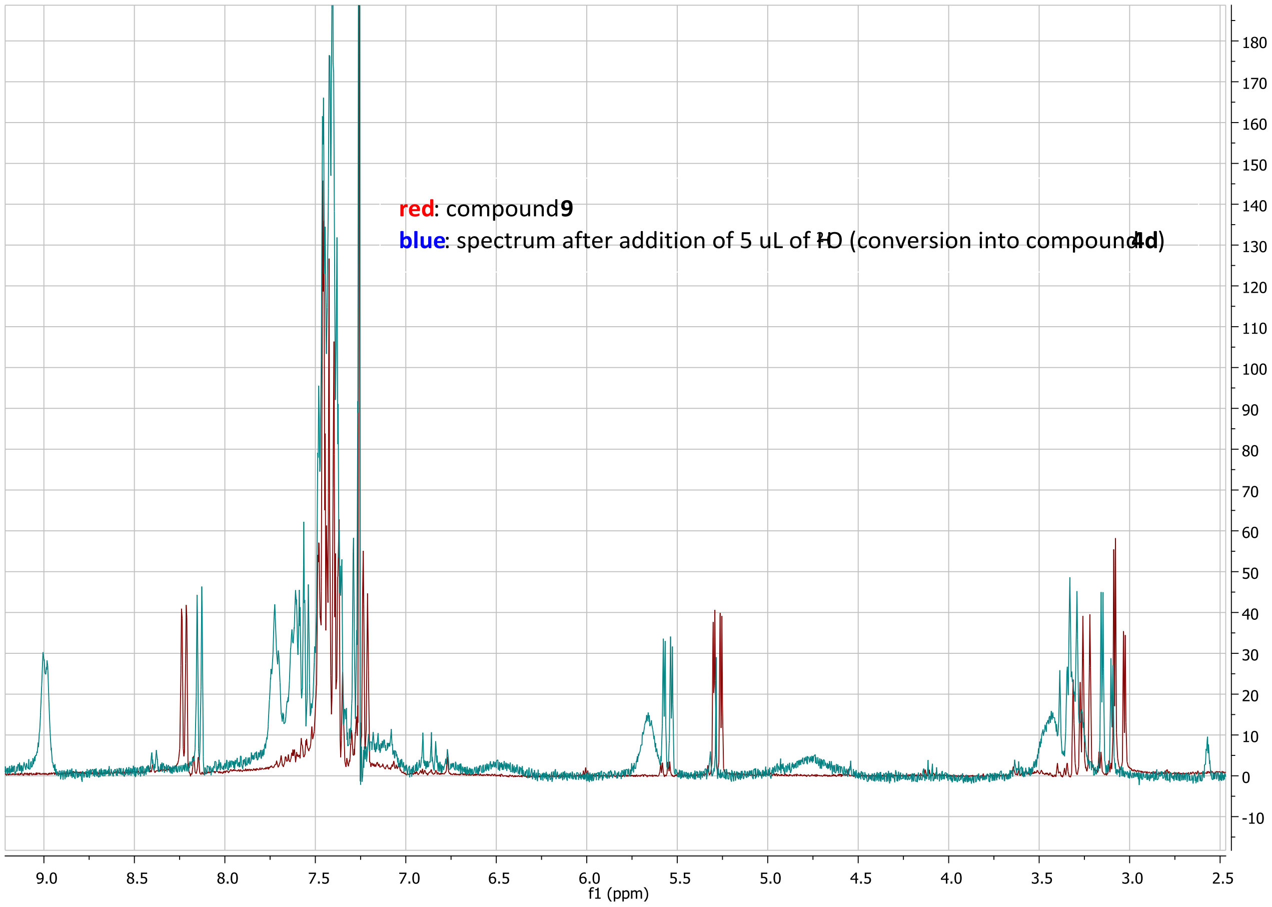

red: compound 9
blue: spectrum after addition of 5 uL of H2O (conversion into compound 4d)
f1 (ppm)

Compound 13a

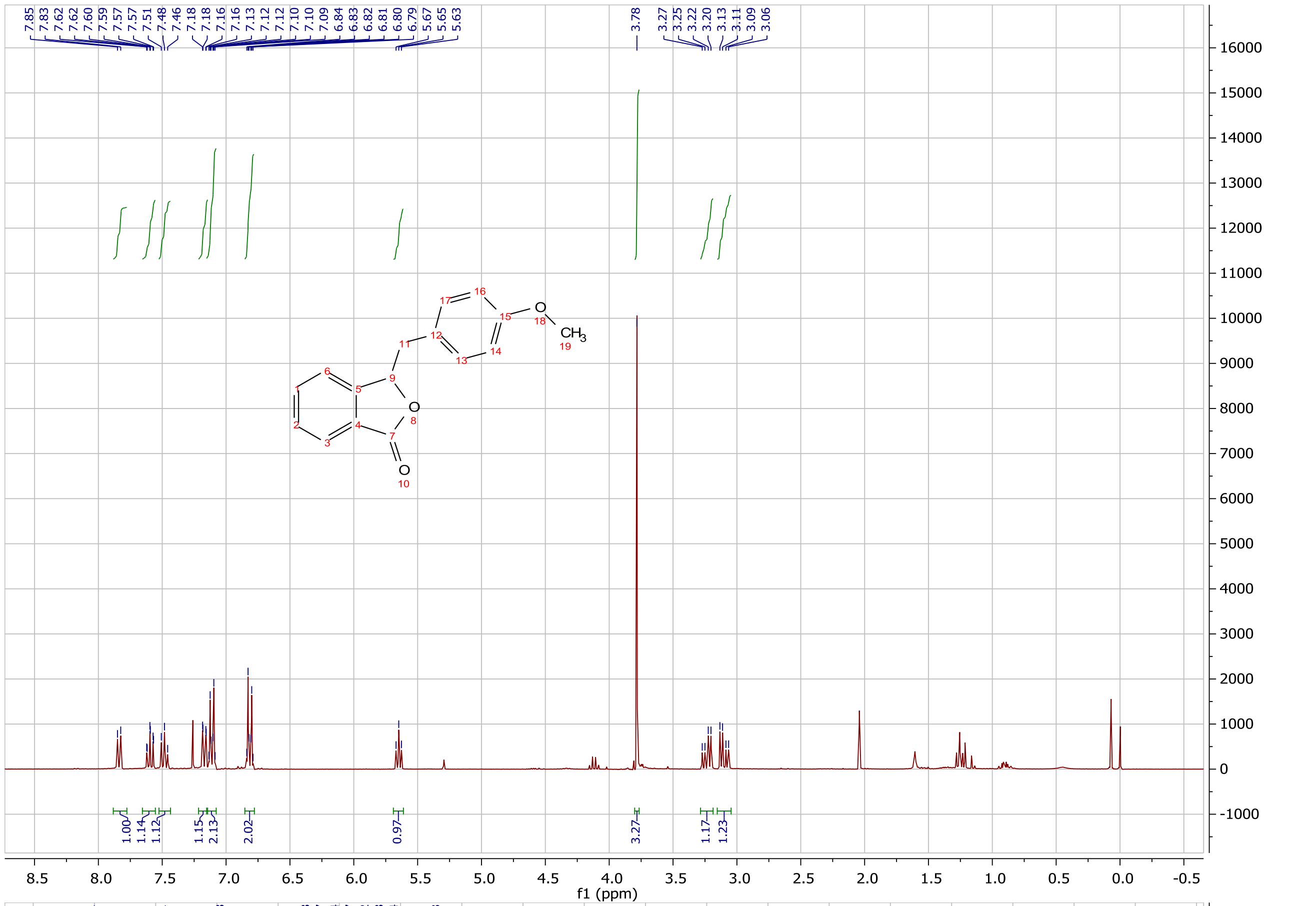

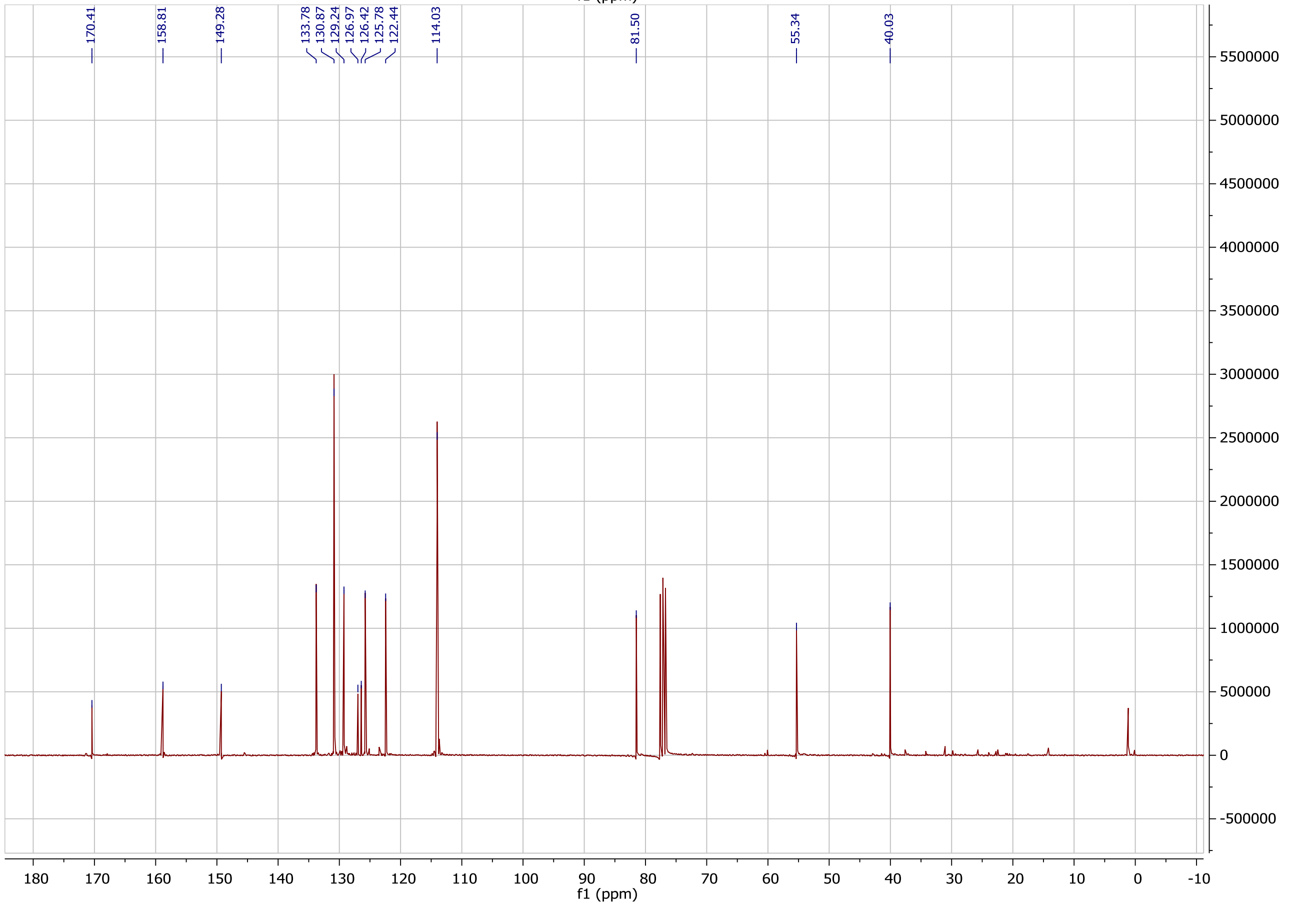

Compound 13b

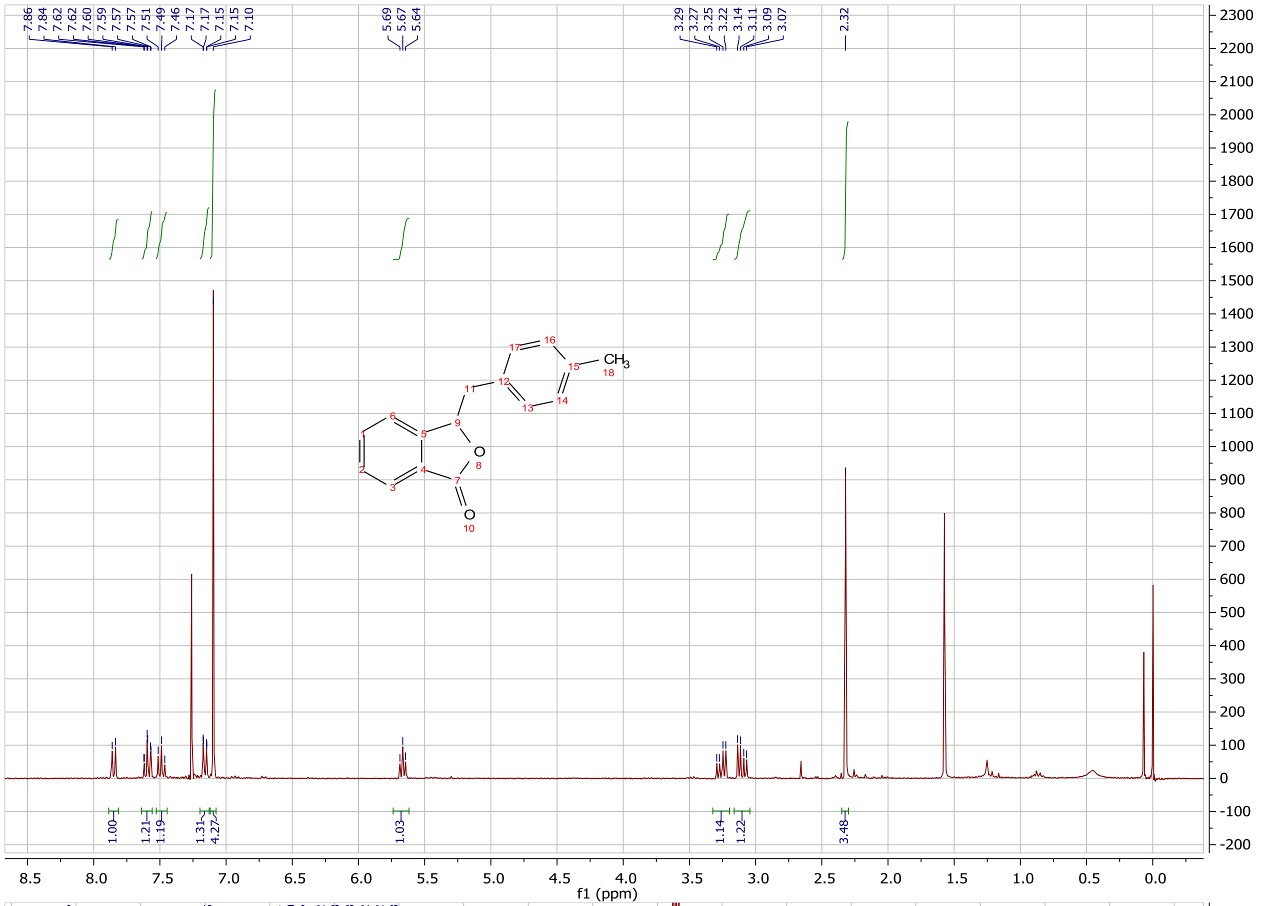

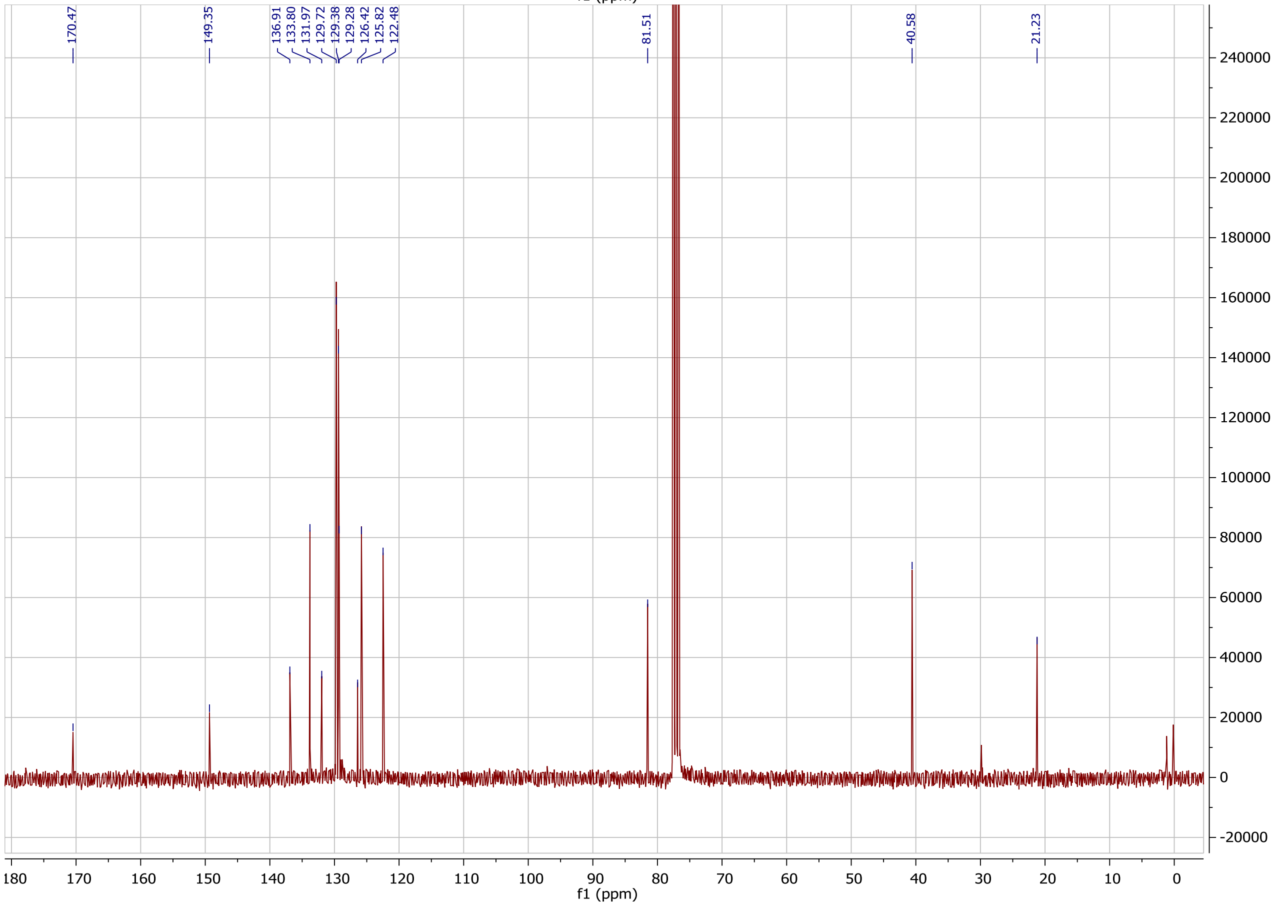

Compound 13c

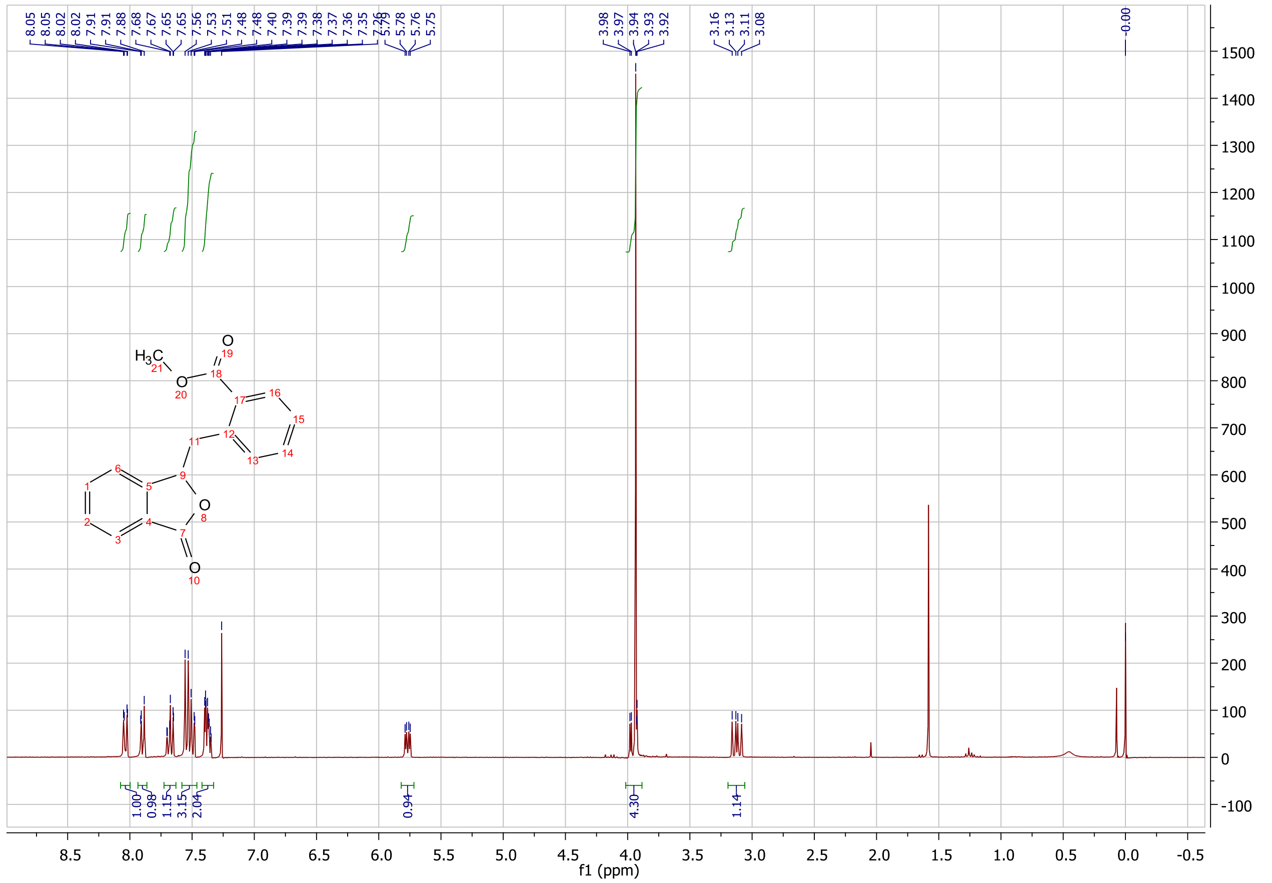

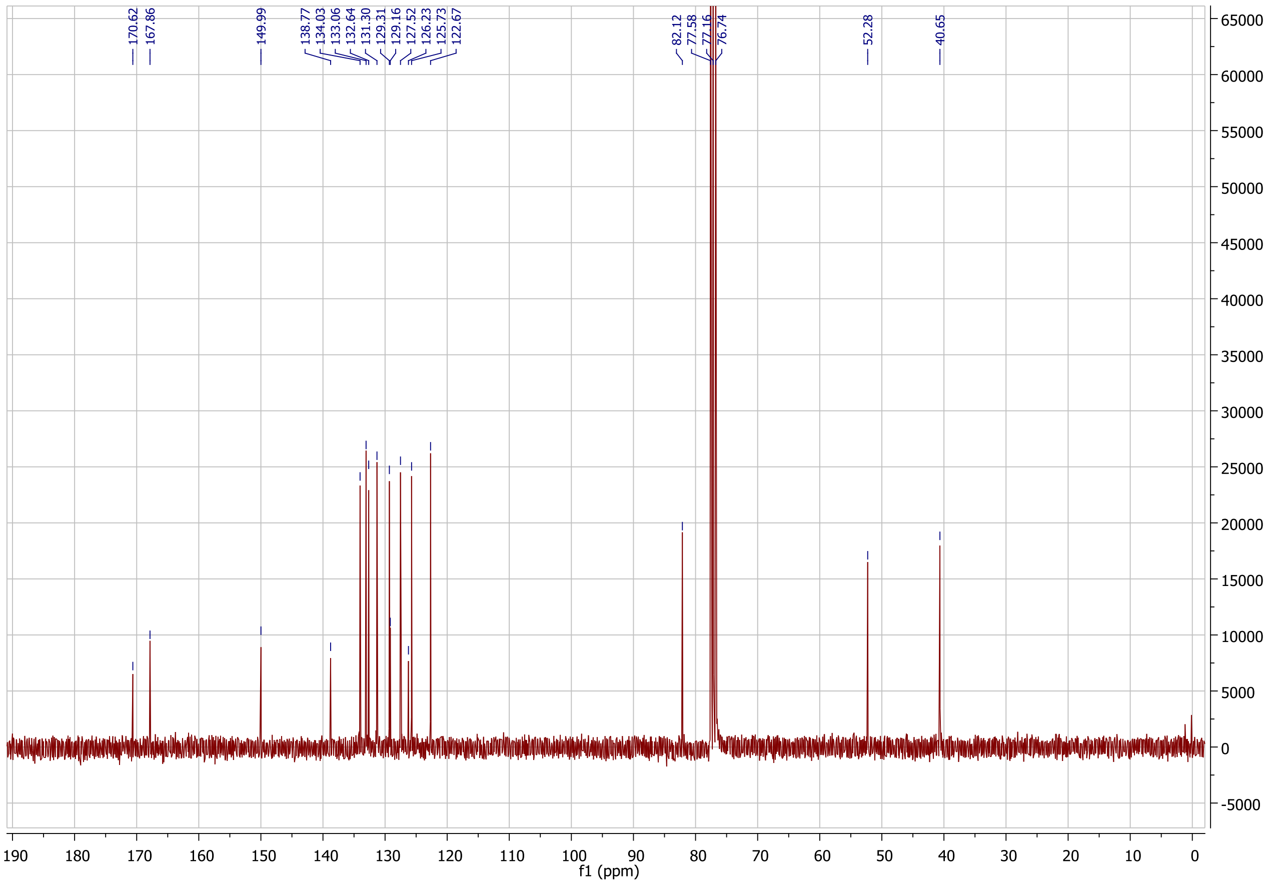

Compound 14a

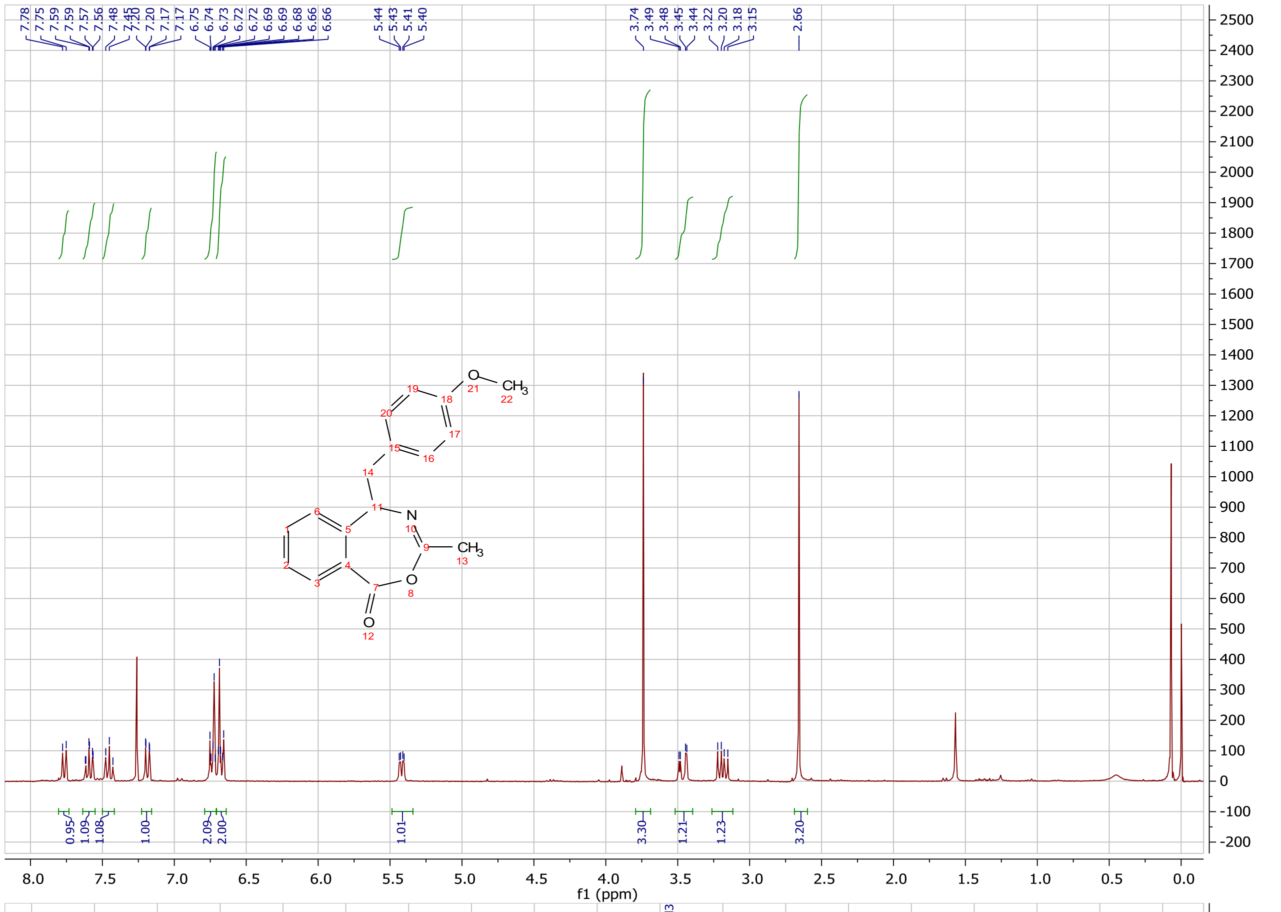

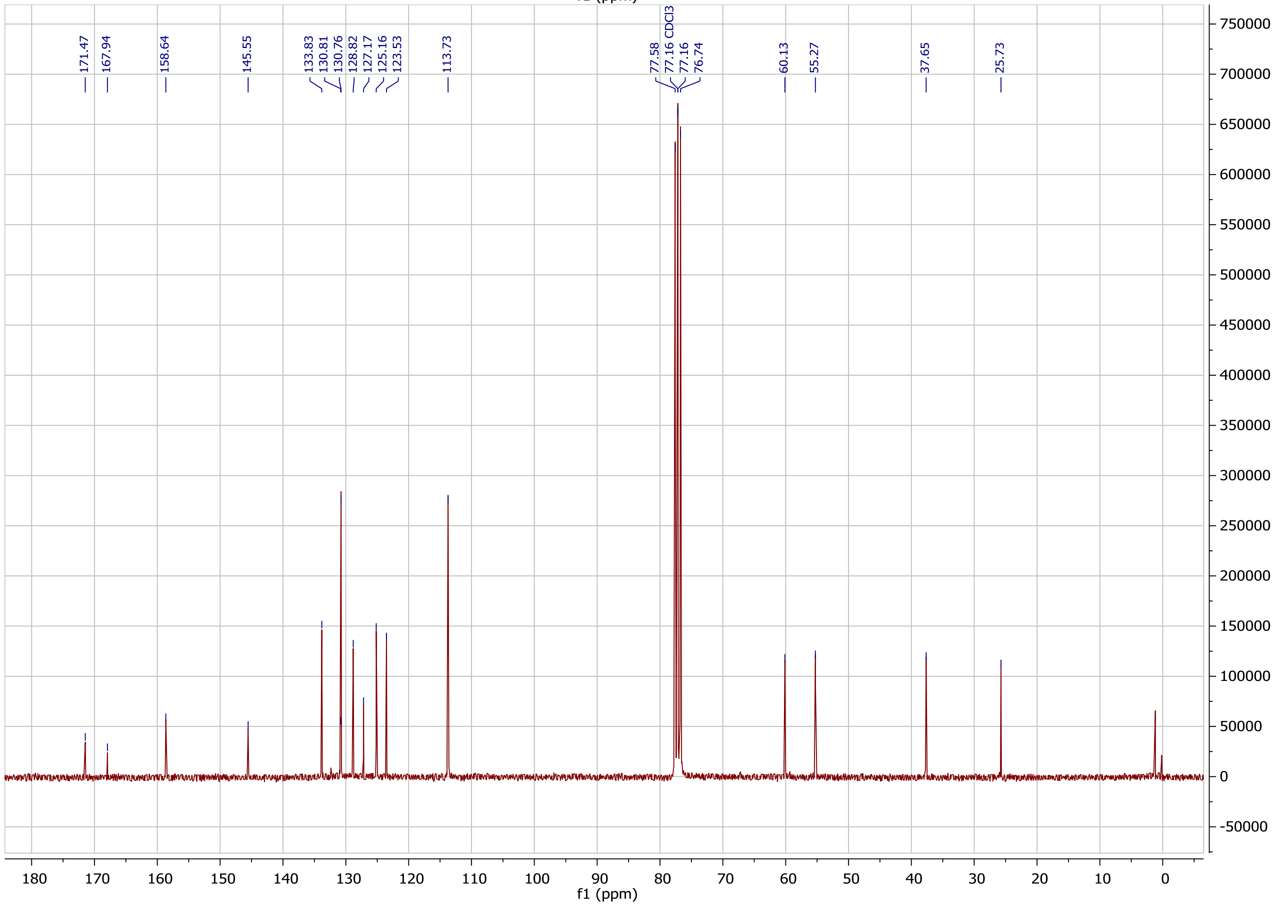

Compound 14b

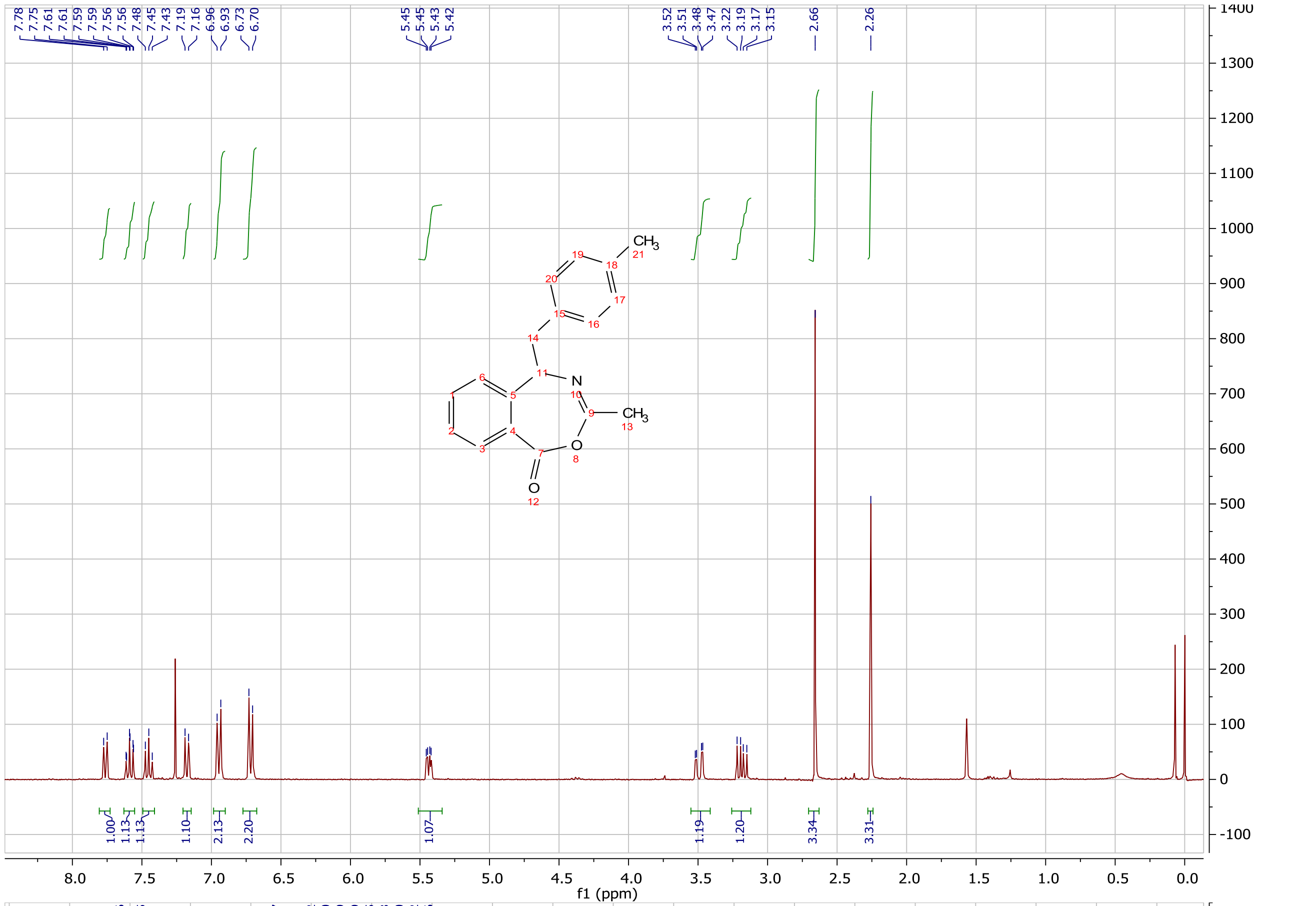

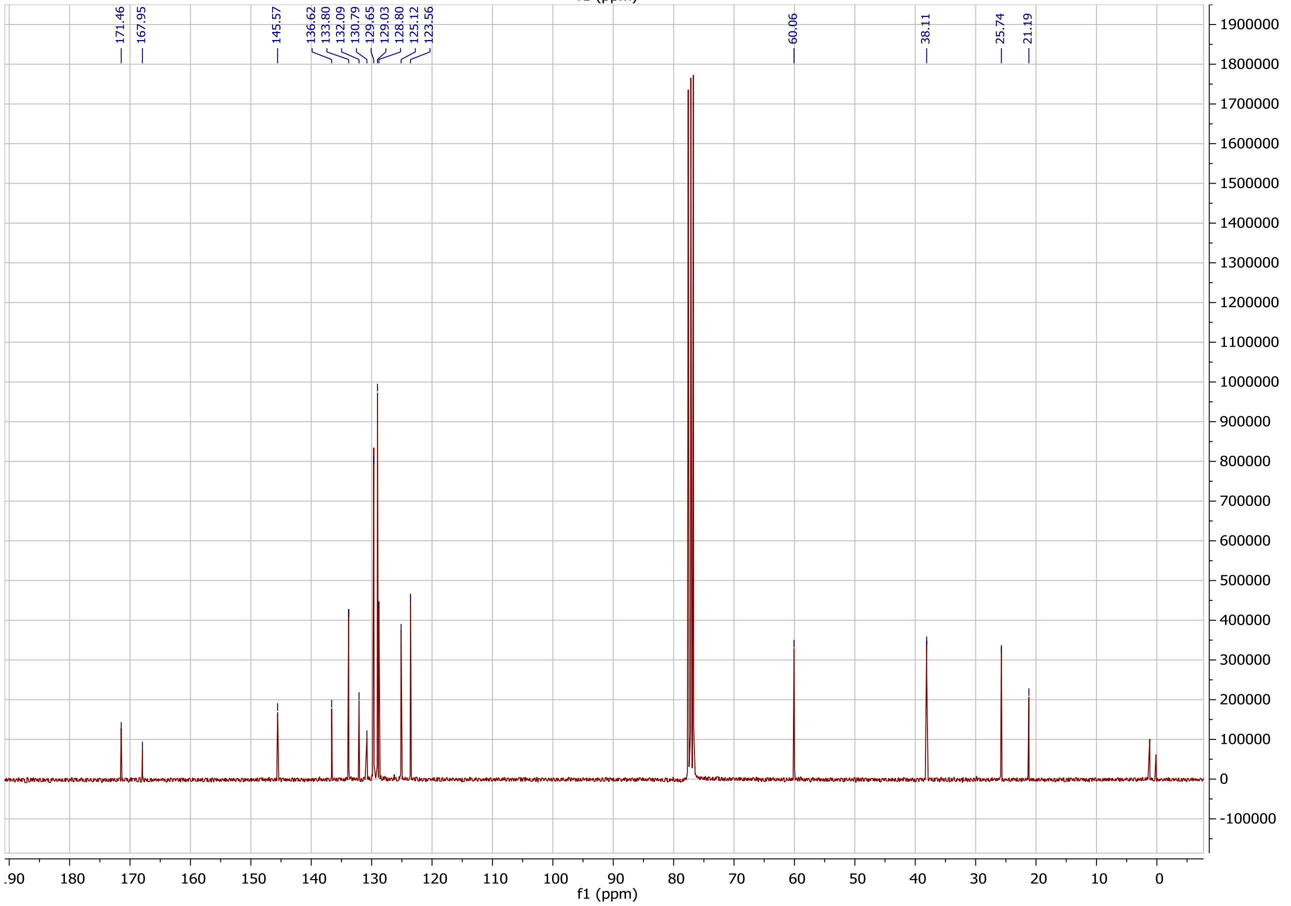

Supplement: File 1 — Experimental details and detailed spectroscopic data. [file Beilstein_J_Org_Chem-13-1456-s001.pdf]
